# New 8-Hydroxybriaranes from the Gorgonian Coral *Junceella fragilis* (Ellisellidae)

**DOI:** 10.3390/md17090534

**Published:** 2019-09-14

**Authors:** You-Ying Chen, Lee-Shing Fang, Yu-Hsin Chen, Bo-Rong Peng, Tung-Pin Su, Thanh-Hao Huynh, Feng-Yu Lin, Chiung-Chin Hu, Nai-Cheng Lin, Zhi-Hong Wen, Jih-Jung Chen, Chieh-Yu Lee, Jin-Wei Wang, Ping-Jyun Sung

**Affiliations:** 1 Department of Marine Biotechnology and Resources, National Sun Yat-sen University, Kaohsiung 804, Taiwan; zoeblack0108@gmail.com (Y.-Y.C.); wzh@mail.nsysu.edu.tw (Z.-H.W.); 2 National Museum of Marine Biology and Aquarium, Pingtung 944, Taiwan; kb5634@yahoo.com.tw (Y.-H.C.); pengpojung@gmail.com (B.-R.P.); g3xz84120@yahoo.com.tw (T.-P.S.); haohuynh0108@gmail.com (T.-H.H.); fengyu30658252@gmail.com (F.-Y.L.); smallsmallhu@gmail.com (C.-C.H.); lnc7222@gmail.com (N.-C.L.); 3 Center for Environmental Toxin and Emerging-Contaminant Research, Cheng Shiu University, Kaohsiung 833, Taiwan; lsfang@csu.edu.tw; 4 Super Micro Mass Research and Technology Center, Cheng Shiu University, Kaohsiung 833, Taiwan; 5 Doctoral Degree Program in Marine Biotechnology, National Sun Yat-sen University, Kaohsiung 804, Taiwan; 6 Doctoral Degree Program in Marine Biotechnology, Academia Sinica, Taipei 115, Taiwan; 7 Graduate Institute of Marine Biology, National Dong Hwa University, Pingtung 944, Taiwan; 8 Department of Applied Chemistry, National Pingtung University, Pingtung 900, Taiwan; 9 Faculty of Pharmacy, School of Pharmaceutical Sciences, National Yang-Ming University, Taipei 112, Taiwan; chenjj@ym.edu.tw; 10 Department of Orthopaedics, Kaohsiung Armed Forces General Hospital, Kaohsiung 802, Taiwan; 11 Chinese Medicine Research and Development Center, China Medical University Hospital, Taichung 404, Taiwan; 12 Graduate Institute of Natural Products, Kaohsiung Medical University, Kaohsiung 807, Taiwan

**Keywords:** *Junceella fragilis*, fragilide, briarane, anti-inflammatory, iNOS

## Abstract

Three new 8-hydroxybriaranes—fragilides R–T (**1**–**3**) were obtained from a sea whip gorgonian coral *Junceella fragilis*. The structures of briaranes **1**–**3** were elucidated by using spectroscopic methods, including 1D (^1^H and ^13^C NMR), 2D (COSY, HSQC, HMBC, and NOESY experiments) NMR studies, and (+)-HRESIMS. Fragilides S and T (**2** and **3**) are the only briaranes known to possess 8α-hydroxy and 17β-methyl groups, respectively. Briarane **2** exerted an inhibition effect on iNOS release from RAW264.7; a macrophage cell line that originated from a mouse monocyte macrophage, stimulated with lipopolysaccharides.

## 1. Introduction

**Figure 1:**
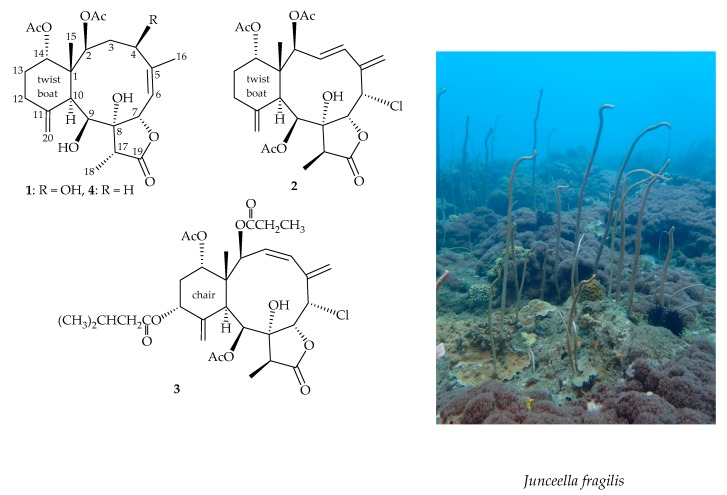
Structures of fragilides R–T (**1**–**3**), 9-*O*-deacetylumbraculolide A (**4**), and a picture of the gorgonian coral *Junceella fragilis*.

Gorgonian corals belonging to the genus *Junceella* (family Ellisellidae) [1,2,3] have been found to produce marine origin briarane-type diterpenoids in abundance [4]. Our recent research into the chemical constituents and properties of a gorgonian coral, *Junceella fragilis* (Ridley 1884) (Figure 1), which was distributed extensively in the waters of Orchid Island (= Lanyu Island), intersection of Kuroshio current and South China Sea surface current, has resulted in the isolation of three new 8- hydroxybriaranes–fragilides R–T (**1**–**3**) (Figure 1). A pro-inflammatory suppression assay was employed to assess the activity of these isolates against the release of inducible nitric oxide synthase (iNOS) from macrophage cells.

## 2. Results and Discussion

**Figure 2:**
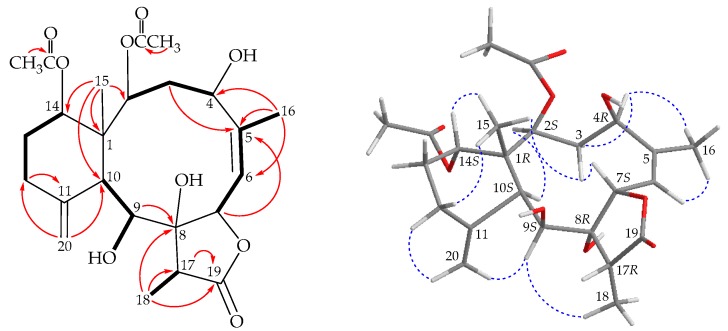
The COSY (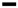) correlations, selective HMBC (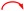), and selective protons with key NOESY correlations (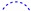) of **1**.

Fragilide R (**1**) was isolated as an amorphous powder and displayed a pseudomolecular ion at *m/z* 489.20971 in the (+)-HRESIMS, which indicated its molecular formula was C_24_H_34_O_9_ (calcd. for C_24_H_34_O_9_ + Na, 489.20950) (Ω = 8). Both the ^1^H and ^13^C NMR data (Table 1 and Table 2) indicated two acetates (δ_H_ 1.99, 1.94, each 3H × s; δ_C_ 21.3, 21.0, 2 × CH_3_; δ_C_ 170.8, 170.8, 2 × C). Besides the above ester carbonyls, the carbon signal at δ_C_ 176.7 (C) was assigned to a γ-lactone ring along with an oxymethine (δ_H_ 5.97, 1H, d, *J* = 10.2 Hz; δ_C_ 76.4, CH-7). The spectroscopic data, including 1D and 2D NMR experiments (Figure 2), were similar to those of a known metabolite, 9-*O*-deacetyl- umbraculolide A (**4**) [5] (Figure 1), except that the hydroxy group at C-4 in **1** was replaced by a proton in **4**. It is interesting to note that an allylic coupling was observed between H-6 and H_3_-16 (*J* = 1.2 Hz) in the COSY spectrum (Figure 2). In the NOESY spectrum (Figure 2), one of the C-3 methylene protons (δ_H_ 2.96) exhibited a correlation to H-7 and not with H-2, suggesting the β- orientation of this proton. A correlation from H-4 to H-3α (δ_H_ 1.91) as well as the coupling constants between H-4 and H-3α/β (*J* = 5.4, 12.6 Hz), suggested that H-4 was α-oriented according to modeling study. Based on the above findings, the structure, including the relative configuration of stereogenic centers of **1** were assigned as 1R*,2S*,4R*,7S*,8R*,9S*,10S*,14S*, and 17R*, as those of **4** by correlations observed in a NOESY experiment.

One of the C-20 methylene protons (δ_H_ 4.88) showed an NOE correlation to H-9, demonstrating that this olefin proton was H-20b, and the other was assigned as H-20a (δ_H_ 4.97). The proton chemical shifts of the briarane derivatives containing an 11-methylidene group were summarized, and the difference between these two olefin protons (H-20a/b) was smaller than 0.2 ppm, whereas the methylidene-containing six-membered rings exhibited a twisted boat conformation [6]. Owing to the chemical shifts of C-20 methylene protons (δ_H_ 4.97 and 4.88), the conformation of six- membered ring in **1** was concluded to be twisted boat. In a previous study, the absolute configuration of a known chlorinated briarane, junceellin, was established by a single-crystal X-ray diffraction analysis [7]. As briaranes **1**–**3** were isolated along with junceellin from the same organism [7], it is reasonable on biogenetic grounds to assume that **1**–**3** have the same absolute configuration as that of junceellin. Therefore, the configuration of the stereogenic centers of **1** were elucidated as 1*R*,2*S*,4*R*,7*S*,8*R*,9*S*,10*S*,14*S,* and 17*R* (Figure 2), and this compound was found to be the 4β-hydroxy derivative of **4** (Supplementary Materials, Figures S1–S9). 

Fragilide S (**2**) had a molecular formula C_26_H_33_ClO_9_ as deduced by (+)-ESIMS, which showed a pair of peaks at *m/z* 547/549 [M + Na]^+^:[M + 2 + Na]^+^ (3:1), suggesting a chlorine atom, and further confirmed by (+)-HRESIMS at *m/z* 547.17055 (calcd. for C_26_H_33_^35^ClO_9_ + Na, 547.17053). The IR spectrum indicated the presence of hydroxy (3447 cm^−1^), γ-lactone (1785 cm^−1^), and ester carbonyl (1733 cm^−1^) groups. The ^13^C NMR data (Table 2), showed the presence of a disubstituted olefin (δ_C_ 135.4, CH-4; 129.7, CH-3) and two methylidene groups (δ_C_ 142.6, C-5; 118.8, CH_2_-16; 149.3, C-11; 111.4, CH_2_-20). Moreover, four carbonyl resonances at δ_C_ 174.1, 170.4, 170.1, and 169.2 in the ^13^C spectrum confirmed the presence of a γ-lactone and three other ester groups. In the ^1^H NMR spectrum (Table 1), three acetate methyls (δ_H_ 2.09, 2.04, 2.01, each 3H × s) were observed. Moreover, a methyl singlet, a methyl doublet, two aliphatic methines, two pair of aliphatic methylenes, four oxymethines, a chlorinated methine, and a hydroxy proton were observed (Table 1).

**Figure 3:**
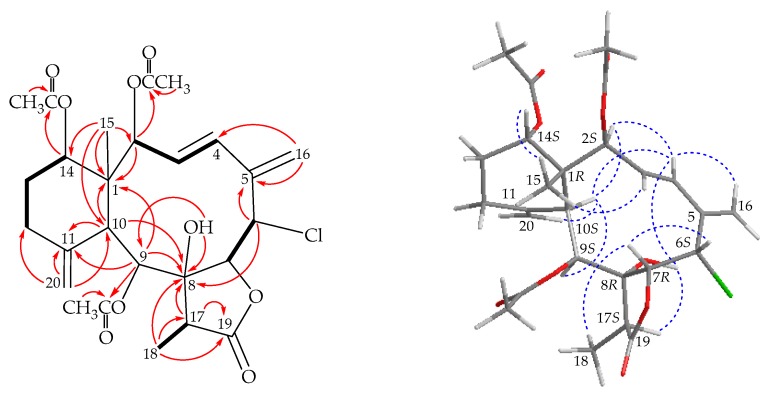
The COSY (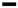) correlations, selective HMBC (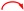), and selective protons with key NOESY correlations (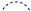) of **2**.

Analyses of 2D NMR data established a tricyclic nucleus. This assignment was evident from the spin systems from H-2 to H-3, H-3 to H-4, H-6 to H-7, H-9 to H-10, H_2_-12 to H_2_-13, H_2_-13 to H-14, and H-17 to H_3_-18 (Figure 3), while the HMBC between protons and quaternary carbons such as H-2, H-9, H-10, H_3_-15/C-1; H-6, H-16a/C-5; H-6, H-9, H-10, H-17, H_3_-18, OH-8/C-8; H-9, H-10, H-20b/C-11; and H-17, H_3_-18/C-19, revealed the carbon skeleton (Figure 3). The methylidene groups at C-5 and C-11 were confirmed by the HMBC between H_2_-16 to C-4 and C-5, H_2_-20 to C-10, C-11, and C-12, respectively. The C-15 methyl group at C-1 was confirmed by the HMBC between H_3_-15 to C-1, C-2, C-10, and C-14. HMBC spectrum also revealed that the carbon signal at δ_C_ 170.4, 170.1, and 169.2 correlated with the signals of the methyl protons at δ_H_ 2.04, 2.01, and 2.09, respectively, and were assigned as the carbon atom of acetate carbonyl groups. The acetates at C-2, C-9, and C-14 were confirmed from the connectivity between H-2 (δ_H_ 5.47), H-9 (δ_H_ 5.53), and H-14 (δ_H_ 4.84) to the carbonyl carbons of the acetate groups at δ_C_ 170.1, 169.2, and 170.4, respectively. The hydroxy group at C-8 was deduced from the HMBC of a hydroxy proton (δ_C_ 3.11) to C-8 and C-9.

In the NOESY spectrum of **2** (Figure 3), one of the C-16 methylene protons (δ_H_ 5.49) showed a correlation to H-4, demonstrating that this olefinic proton was H-16a and the other was assigned as H-16b (δ_H_ 5.32). Moreover, one of the C-20 methylene protons (δ_H_ 4.76) correlated to H-10, indicating that this proton was H-20b and the other was assigned as H-20a. According to a summary for the chemical shifts of 11-methylidene groups, the configuration of six-membered ring was in a twisted boat conformation [6]. The *E*-geometry of C-3/4 double bond was determined by a large proton coupling constant (*J* = 16.0 Hz) between H-3 and H-4. Correlations between H-10 with H-2 and H-9, while no correlation was seen with Me-15, suggested that H-2, H-9, and H-10 were all in α-oriented. Meanwhile, a correlation of Me-15 with H-14 indicated that H-14 was β-oriented. Furthermore, OH-8 showed correlations with H-4 and H-17, indicating that the hydroxy group at C-8 and proton at C-17 were α-oriented. In addition, H-7 exhibited correlations with H-6 and H_3_-18 but not with OH-8, suggesting that H-7 was β-oriented. Based on above findings, the configuration of stereogenic carbons was determined as 1*R*,2*S*,6*S*,7*R*,8*R*,9*S*,10*S*,14*S* and 17*S* (see Figures S10–S18 in the Supplementary Materials).

**Figure 4:**
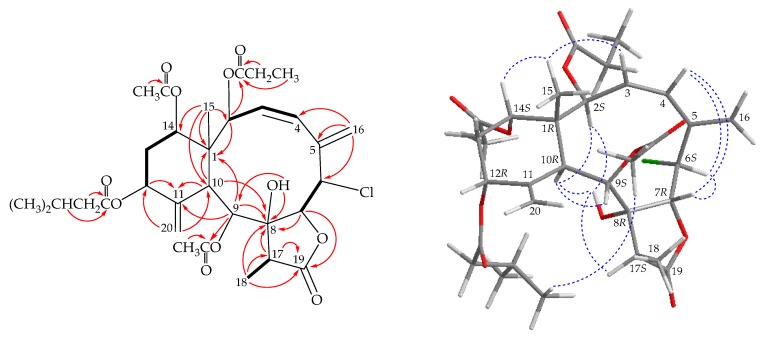
The COSY (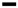) correlations, selective HMBC (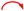), and selective protons with key NOESY correlations (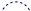) of **3**.

Compound **3** (fragilide T) has a molecular formula C_32_H_43_ClO_11_ according to its (+)-HRESIMS at *m/z* 661.23866 (calcd. for C_32_H_43_^35^ClO_11_ + Na, 661.23861). Both the ^1^H and ^13^C NMR (Table 1 and Table 2) indicated two acetates (δ_H_ 2.16, 2.00, each 3H × s; δ_C_ 21.3, 20.7, 2 × acetate methyls; 169.7, 169.6, 2 × acetate carbonyls), a propionate (δ_H_ 1.08, 3H, t, *J* = 7.2 Hz; 2.26, 2H, m; δ_C_ 8.8, CH_3_; 27.8, CH_2_; 172.0, propionate carbonyl), and an isovalerate (δ_H_ 0.96, 0.98, each 3H, d, *J* = 6.6 Hz; 2.10, 1H, m; 2.15, 2H, m; δ_C_ 22.6, 22.8, 2 × CH_3_; 25.0, CH; 43.5, CH_2_; 172.6, isovalerate carbonyl). Besides the above ester carbonyls, the carbon signal at δ_C_ 173.9 was assigned to a γ-lactone ring along with an oxymethine (δ_H_ 4.88, 1H, d, *J* = 4.2 Hz; δ_C_ 76.9, CH-7). Two pairs of proton signals at δ_H_ 5.66 and 5.48, and 5.33 and 4.72, correlating to the methylidene signals at δ_C_ 116.4 and 115.2 respectively, were ascribed to two methylidene groups. The tertiary methyl singlet at δ_H_ 1.16 (3H, s) was assigned to H_3_-15 while the secondary methyl doublet at δ_H_ 1.22 (3H, d, *J* = 7.2 Hz) was assigned to H_3_-18. In the HMBC spectrum (Figure 4), the propionoxy group at C-2 was confirmed by the connectivity between H-2 (δ_H_ 6.33) with the carbonyl carbon (δ_C_ 172.0) of propionoxy group. The HMBC also revealed that an acetoxy group at C-9 (Figure 4) and the remaining isovaleroxy and acetoxy groups should be positioned at C-12 or C-14, oxygen-bearing methines, by analysis of characteristic NMR signals (δ_H_ 5.37, 1H, dd, *J* = 5.4, 3.0 Hz; δ_C_ 74.3, CH-12; δ_H_ 4.94, 1H, dd, *J* = 4.2, 3.0 Hz; δ_C_ 73.0, CH-14). However, due to no HMBC detected between H-12 and H-14 and ester carbonyl, the positions of the isovalerate and remaining acetoxy group cannot be determined by HMBC.

Based on previous studies, while the difference between the two olefin protons (H-20a/b) was bigger than 0.3 ppm, the six-membered rings showed a chair conformation [6]. Owing to the chemical shifts of the C-20 methylene protons (δ_H_ 5.33 and 4.72 ppm), the configuration of the methylidene-containing six-membered ring was concluded to exist in a chair conformation. In the NOESY experiment (Figure 4), H-10 correlated to H-2, H-9, and OH-8, but not to H_3_-15, indicating that these protons are located on the same face and can be assigned as α-protons, as C-15 methyl group is a β-substituent at C-1. H-14 was found to exhibit a correlation with H_3_-15, showing that this proton is positioned on the equatorial direction and has a β-orientation at C-14. The *cis* geometry of the C-3/4 double bond was indicated by a 12.0 Hz coupling constant between H-3 (δ_H_ 5.78) and H-4 (δ_H_ 5.96). Moreover, a correlation between H-3 and H_3_-15, and there are correlations which were observed among H-4, H-6, and H-7, further supported the *Z*-form of C-3/4 double bond and indicated that H-6 and H-7 were on the β face. A correlation between OH-8 and H-17 showed that Me-18 at C-17 was β-oriented. The C-12 oxymethine proton (δ_H_ 5.37) was found to couple C-13 methylene protons with coupling constants *J* = 5.4, 3.0 Hz, showing that this proton should be positioned on the equatorial direction and has a β-orientation. Fortunately, a correlation between the methyl protons of acetoxy group at C-9 and methyl protons of isovaleroxy group, indicated that the isovaleroxy group should be located at C-12 by modeling analysis. Thus, based on the above findings, the stereogenic centers were assigned as 1*R*,2*S*,6*S*,7*R*,8*R*,9*S*,10*R*,12*R*,14*S,* and 17*S* (see Figures S19–S27 in the Supplementary Materials).

Using an *in vitro* pro-inflammatory suppression assay, the effects of briaranes **1**–**3** on the release of inducible nitric oxide synthase (iNOS) and cyclooxygenase-2 (COX-2) protein from lipopolysaccharides (LPS)-stimulated RAW264.7 macrophage cells were assessed. The results of pro-inflammatory suppression assay showed that briarane **2** at 10 μM suppressed the release of iNOS to 61.21 ± 9.61% as compared with results of the cells stimulated with LPS only (Table 3). 

## 3. Materials and Methods

### 3.1. General Experimental Procedures

NMR spectra were recorded on a 600 MHz Jeol NMR (model ECZ600R, Tokyo, Japan) or on 500 MHz Varian NMR (model Unity Inova-500, Palo Alto, CA, USA) spectrometers using the residual CHCl_3_ signal (δ_H_ 7.26 ppm) and CDCl_3_ (δ_C_ 77.1 ppm) as internal references for ^1^H and ^13^C NMR, respectively. ESIMS and HRESIMS were obtained from the Bruker mass spectrometer with 7 Tesla magnets (model: SolariX FTMS system, Bremen, Germany). Column chromatography, high-performance liquid chromatography (HPLC), IR spectra, and optical rotation were performed according to our earlier research [7]. 

### 3.2. Animal Material

Specimens of *J. fragilis* used for this study were collected in June 2017 by self-contained underwater breathing apparatus (SCUBA) divers off the coast of Orchid Island, Taiwan. The samples were stored in a −20 °C freezer until extraction. A voucher specimen was deposited in the NMMBA (voucher no.: NMMBA-TW-GC-2017-08). Identification of the species of this organism was performed by comparison as described in previous studies [1,2,3].

### 3.3. Extraction and Isolation

Sliced bodies (wet weight = 423 g) of the coral specimen were prepared and extracted with a 1:1 mixture of methanol (MeOH) and dichloromethane (CH_2_Cl_2_) to give 5.53 g of crude extract, which was partitioned between ethyl acetate (EtOAc) and H_2_O. The EtOAc extract (2.50 g) was then applied to a silica gel column and eluted with gradients of *n*-hexane/acetone (stepwise from 50:1 to 1:2; volume ratio) to furnish 8 fractions (fractions: A−H). Fraction F was purified by normal-phase HPLC (NP-HPLC) using a mixture of *n*-hexane and EtOAc (3.5:1 of volume ratio) as solvent to obtain 14 subfractions (fractions: F1−F14). Fraction F10 was repurified by reverse-phase HPLC (RP-HPLC) using a mixture of MeOH and H_2_O (with volume:volume = 80:20; at a flow rate = 4.0 mL/min) to yield **2** (0.2 mg) and **3** (0.5 mg). Fraction G was separated by NP-HPLC using a mixture of *n*-hexane/EtOAc (1:1; volume ratio) to yield 9 fractions (fractions: G1−G9). Fraction G2 was purified by RP-HPLC using a mixture of MeOH and H_2_O (with volume:volume = 65:35; at a flow rate = 4.0 mL/min) to afford **1** (0.2 mg). 

Fragilide R (**1**): Amorphous powder; [α]$\overset{28}{\underset{D}{}}$ −288 (*c* 0.07, CHCl_3_); IR (ATR) ν_max_ 3391, 1779, 1736 cm^−1^; ^1^H (600 MHz, CDCl_3_) and ^13^C (150 MHz, CDCl_3_) NMR data, see Table 1 and Table 2; ESIMS: *m/z* 489 [M + Na]^+^; HRESIMS: *m/z* 489.20971 (calcd. for C_24_H_34_O_9_ + Na, 489.20950).

Fragilide S (**2**): Amorphous powder; [α]$\overset{28}{\underset{D}{}}$ +45 (*c* 0.16, CHCl_3_); IR (ATR) ν_max_ 3447, 1785, 1733 cm^−1^; ^1^H (500 MHz, CDCl_3_) and ^13^C (125 MHz, CDCl_3_) NMR data, see Table 1 and Table 2; ESIMS: *m/z* 547 [M + Na]^+^, 549 [M + 2 + Na]^+^; HRESIMS: *m/z* 547.17055 (calcd. for C_26_H_33_^35^ClO_9_ + Na, 547.17053).

Fragilide T (**3**): Amorphous powder; [α]$\overset{20}{\underset{D}{}}$ −168 (*c* 0.17, CHCl_3_); IR (ATR) ν_max_ 1788, 1737 cm^−1^; ^1^H (600 MHz, CDCl_3_), and ^13^C (150 MHz, CDCl_3_) NMR data, see Table 1 and Table 2; ESIMS: *m/z* 661 [M + Na]^+^, 663 [M + 2 + Na]^+^; HRESIMS: *m/z* 661.23866 (calcd. for C_32_H_43_^35^ClO_11_ + Na, 661.23861).

### 3.4. In Vitro Anti-inflammatory Assay

The pro-inflammatory suppression assay was employed to assess the activities of the isolated compounds **1**–**3** against the release of iNOS and COX-2 from macrophage cells as the literature reported [8, 9, 10, 11].

## 4. Conclusions

*J. fragilis* has been demonstrated to have a wide structural diversity of briarane-type diterpenoids that possess various pharmacological properties, particularly in anti-inflammatory activity [12,13]. In our continued study of *J. fragilis*, three previously unreported fragilides R–T (**1**–**3**) were isolated. In the previous studies [14], all the Me-18 attached at C-17 was *cis* to the hydroxy group at C-8, and most of these two groups were α-oriented in briarane derivatives, respectively. Fragilides S and T (**2** and **3**) were proved to be the only two briaranes known to possess hydroxy group at C-8α and methyl group at C-17β, respectively. In the present study, the anti-inflammatory activity of **1**–**3** was assessed using inhibition of pro-inflammatory iNOS and COX-2 release from macrophages. The results indicated that fragilide S (**2**) showed the most potent suppressive effect on iNOS release.

## Figures and Tables

**Table 1:** ^1^H NMR data for briaranes **1**–**3**.

Position	1 ^a^	2 ^b^	3 ^a^
2	4.83 d (7.2) ^c^	5.47 d (9.5)	6.33 d (10.8)
3α/β	1.91 ddd (15.6, 7.2, 5.4); 2.96 dd (15.6, 12.6)	5.67 dd (16.0, 9.5)	5.78 dd (12.0, 10.8)
4	4.18 dd (12.6, 5.4)	6.72 d (16.0)	5.96 d (12.0)
6	5.64 d (10.2, 1.2)	5.20 s	5.12 br s
7	5.97 d (10.2)	4.97 br s	4.88 d (4.2)
9	4.22 dd (5.4, 3.6)	5.53 s	5.59 s
10	3.20 d (3.6)	3.47 s	4.08 s
12α/β	2.22 m	2.24 m; 2.38 m	5.37 dd (5.4, 3.0)
13α/β	1.96 m; 1.81 m	1.84 m; 1.74 m	2.06 m
14	4.74 dd (4.2, 1.8)	4.84 dd (2.5, 2.0)	4.94 dd (4.2, 3.0)
15	1.25 s	1.18 s	1.16 s
16a/b	2.09 d (1.2)	5.49 s; 5.32 s	5.66 s; 5.48 s
17	3.11 q (7.2)	2.93 q (7.5)	2.90 q (7.2)
18	1.14 d (7.2)	1.18 d (7.5)	1.22 d (7.2)
20a/b	4.97 s; 4.88 s	4.93 s; 4.76 s	5.33 s; 4.72 s
OH-8	-	3.11 s	2.70 s
OH-9	2.09 d (5.4)	-	-
Acetoxy groups	1.94 s 1.99 s	2.01 s 2.04 s 2.09 s	2.00 s 2.16 s
Propionoxy group	-	-	1.08 t (7.2) 2.26 m
Isovaleroxy group	-	-	0.96 d (6.6) 0.98 d (6.6) 2.10 m 2.15 m

^a^ Spectra measured at 600 MHz in CDCl_3_. ^b^ Spectra measured at 500 MHz in CDCl_3_. ^c^ *J* values (in Hz) in parentheses.

**Table 2:** ^13^C NMR data for briaranes **1**–**3**.

Position	1 ^a^	2 ^b^	3 ^a^
1	48.2, C ^c^	47.5, C	47.7, C
2	73.3, CH	76.9, CH	70.4, CH
3	39.7, CH_2_	129.7, CH	130.4, CH
4	71.4, CH	135.4, CH	128.7, CH
5	146.9, C	142.6, C ^d^	137.5, C
6	123.1, CH	66.0, CH ^d^	64.5, CH
7	76.4, CH	79.5, CH	76.9, CH
8	83.4, C	81.8, C	80.3, C
9	74.1, CH	76.3, CH	74.8, CH
10	43.0, CH	41.8, CH	37.0, CH
11	152.3, C	149.3, C	146.3, C
12	29.0, CH_2_	29.1, CH_2_	74.3, CH
13	27.8, CH_2_	26.0, CH_2_	31.4, CH_2_
14	74.2, CH	74.4, CH	73.0, CH
15	15.6, CH_3_	16.4, CH_3_	14.7, CH_3_
16	26.2, CH_3_	118.8, CH_2_^d^	116.4, CH_2_
17	43.8, CH	49.3, CH	50.7, CH
18	6.6, CH_3_	9.1, CH_3_	8.7, CH_3_
19	176.7, C	174.1, C ^d^	173.9, C
20	111.3, CH_2_	111.4, CH_2_	115.2, CH_2_
Acetoxy groups	21.0, CH_3_ 170.8, C 21.3, CH_3_ 170.8, C	21.2, CH_3_ 170.4, C 21.2, CH_3_ 170.1, C 21.2, CH_3_ 169.2, C	20.7, CH_3_ 169.7, C 21.3, CH_3_ 169.6, C
Propionoxy group	-	-	8.8, CH_3_ 27.8, CH_2_ 172.0, C
Isovaleroxy group	-	-	22.6, CH_3_ 22.8, CH_3_ 25.0, CH 43.5, CH_2_ 172.6, C

^a^ Spectra measured at 150 MHz in CDCl_3_. ^b^ Spectra measured at 125 MHz in CDCl_3_. ^c^ Multiplicity deduced by DEPT and HSQC spectra. ^d^ Chemical shfits were assigned by HSQC or HMBC experiments.

**Table 3:** Effects of briaranes **1**–**3** on LPS-induced pro-inflammatory iNOS and COX-2 protein expression in macrophages.

Compound	iNOS	COX-2	β-Actin	*n*
	Expression (% of LPS)			
Negative Control	1.80 ± 0.21	1.04 ± 0.35	110.02 ± 5.23	2
LPS	100.01 ± 5.06	100.06 ± 0.43	100.07 ± 8.4	4
**1**	104.11 ± 16.63	100.51 ± 6.11	105.70 ± 7.05	4
**2**	61.21 ± 9.61	100.01 ± 5.11	99.29 ± 11.29	3
**3**	100.91 ± 24.08	96.36 ± 21.31	115.29 ± 3.4	4
Dexamethasone	5.54 ± 1.72	8.15 ± 5.13	105.21 ± 15.57	4

Data were normalized to those of cells treated with LPS alone, and cells treated with dexamethasone were used as a positive control. Data are expressed as the mean ± SEM (*n* = 2–4).

## Supplementary Materials

The Supplementary Materials are available online at https://www.mdpi.com/1660-3397/17/9/534/s1. Figure S1: ESIMS spectrum of compound 1, Figure S2: HRESIMS spectrum of compound 1, Figure S3: IR spectrum of compound 1, Figure S4: 1H NMR spectrum (600 MHz) of compound 1 in CDCl3, Figure S5: 13C NMR spectrum (150 MHz) of compound 1 in CDCl3, Figure S6: HSQC spectrum of compound 1 in CDCl3, Figure S7: 1H-1H COSY spectrum of compound 1 in CDCl3, Figure S8: HMBC spectrum of compound 1 in CDCl3, Figure S9: NOESY spectrum of compound 1 in CDCl3, Figure S10: ESIMS spectrum of compound 2, Figure S11: HRESIMS spectrum of compound 2, Figure S12: IR spectrum of compound 2, Figure S13: 1H NMR spectrum (600 MHz) of compound 2 in CDCl3, Figure S14: 13C NMR spectrum (150 MHz) of compound 2 in CDCl3, Figure S15: HSQC spectrum of compound 2 in CDCl3, Figure S16: 1H-1H COSY spectrum of compound 2 in CDCl3, Figure S17: HMBC spectrum of compound 2 in CDCl3, Figure S18: NOESY spectrum of compound 2 in CDCl3, Figure S19: ESIMS spectrum of compound 3, Figure S20: HRESIMS spectrum of compound 3, Figure S21: IR spectrum of compound 3, Figure S22: 1H NMR spectrum (600 MHz) of compound 3 in CDCl3, Figure S23: 13C NMR spectrum (150 MHz) of compound 3 in CDCl3, Figure S24: HSQC spectrum of compound 3 in CDCl3, Figure S25: 1H-1H COSY spectrum of compound 2 in CDCl3, Figure S26: MBC spectrum of compound 3 in CDCl3, Figure S27: NOESY spectrum of compound 3 in CDCl3.
