# Supplementary material for: New 8-Hydroxybriaranes from the Gorgonian Coral Junceella fragilis (Ellisellidae)
Source: Mar Drugs. 2019 Sep 14;17(9):534. doi: 10.3390/md17090534 (PMC6780648; doi:10.3390/md17090534)
Supplement: Supplementary file 1 [file marinedrugs-17-00534-s001.pdf]

Supporting Information

No.	Content	Page
S1.	ESIMS spectrum of compound **1**.	2
S2.	HRESIMS spectrum of compound **1**.	2
S3.	IR spectrum of compound **1**.	3
S4.	^{1}H NMR spectrum (600 MHz) of compound **1** in $CDCl_3$.	3
S5.	^{13}C NMR spectrum (150 MHz) of compound **1** in $CDCl_3$.	4
S6.	HSQC spectrum of compound **1** in $CDCl_3$.	4
S7.	^{1}H–^{1}H COSY spectrum of compound **1** in $CDCl_3$.	5
S8.	HMBC spectrum of compound **1** in $CDCl_3$.	5
S9.	NOESY spectrum of compound **1** in $CDCl_3$.	6
S10	ESIMS spectrum of compound **2**.	6
S11.	HRESIMS spectrum of compound **2**.	7
S12.	IR spectrum of compound **2**.	7
S13.	^{1}H NMR spectrum (600 MHz) of compound **2** in $CDCl_3$.	8
S14.	^{13}C NMR spectrum (150 MHz) of compound **2** in $CDCl_3$.	9
S15.	HSQC spectrum of compound **2** in $CDCl_3$.	10
S16.	^{1}H–^{1}H COSY spectrum of compound **2** in $CDCl_3$.	10
S17.	HMBC spectrum of compound **2** in $CDCl_3$.	11
S18.	NOESY spectrum of compound **2** in $CDCl_3$.	11
S19.	ESIMS spectrum of compound **3**.	12
S20.	HRESIMS spectrum of compound **3**.	12
S21.	IR spectrum of compound **3**.	13
S22.	^{1}H NMR spectrum (600 MHz) of compound **3** in $CDCl_3$.	13
S23.	^{13}C NMR spectrum (150 MHz) of compound **3** in $CDCl_3$.	14
S24.	HSQC spectrum of compound **3** in $CDCl_3$.	15
S25.	^{1}H–^{1}H COSY spectrum of compound **2** in $CDCl_3$.	15
S26.	HMBC spectrum of compound **3** in $CDCl_3$.	16
S27.	NOESY spectrum of compound **3** in $CDCl_3$.	16

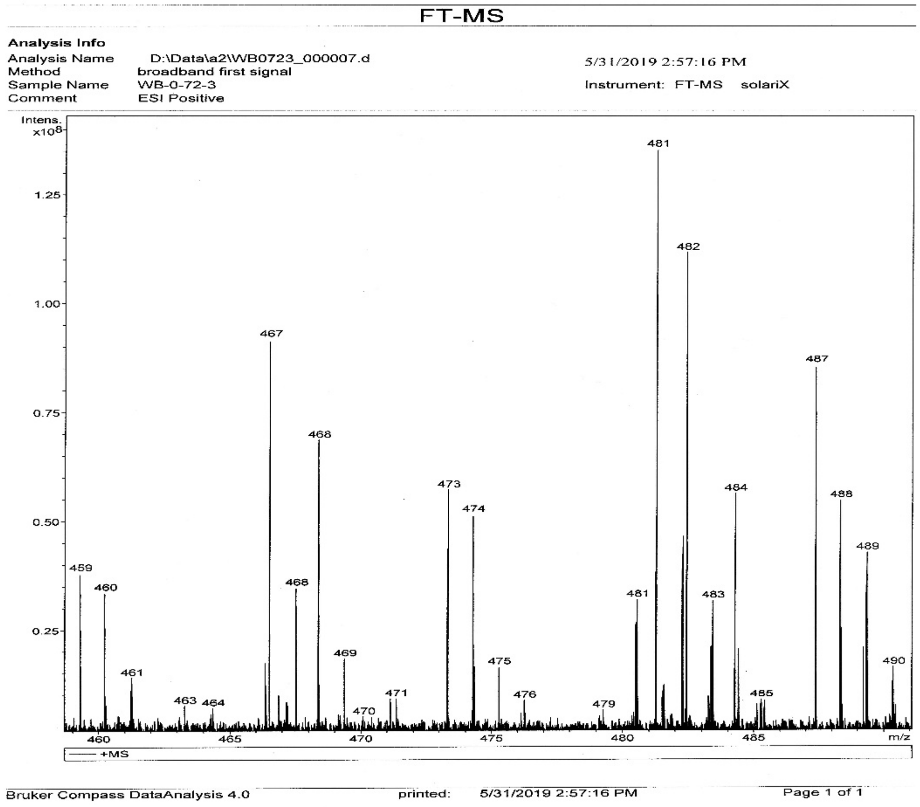

S1. ESIMS spectrum of compound **1**.

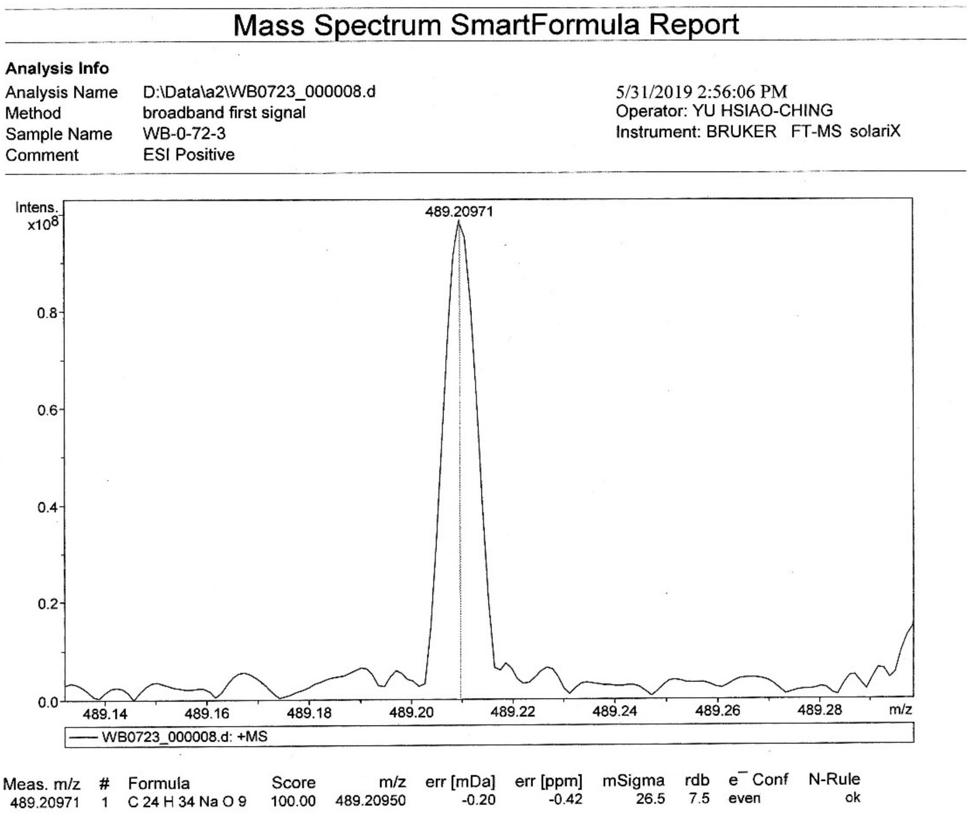

S2. HRESIMS spectrum of compound **1**.

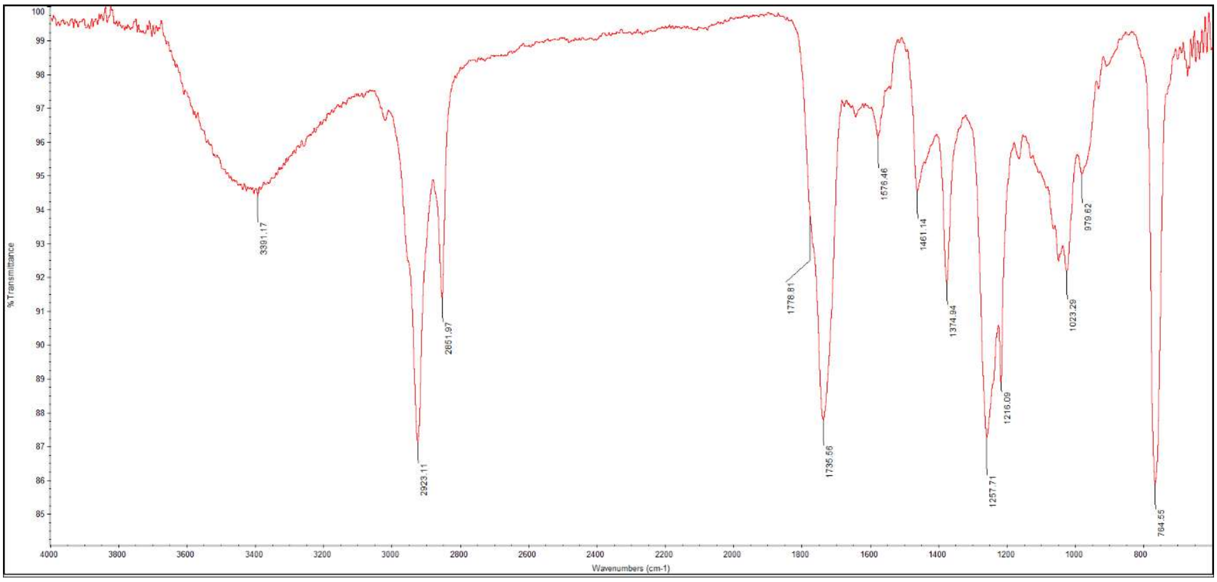

S3. IR spectrum of compound **1**.

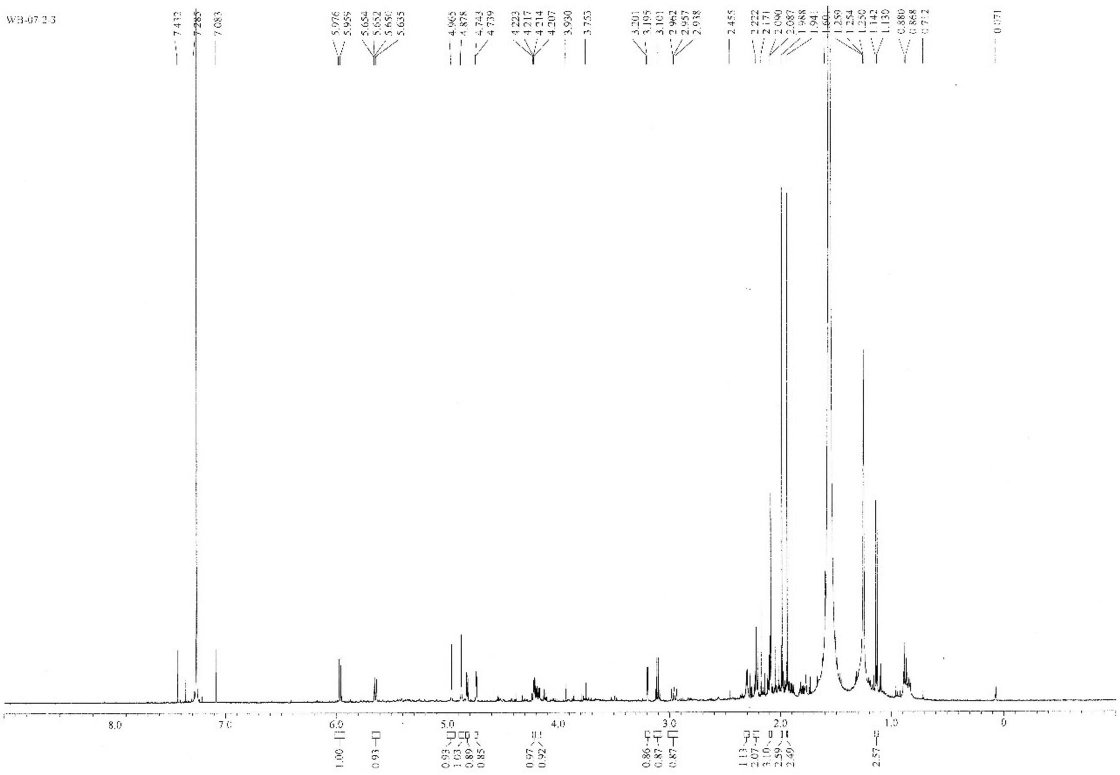

S4. ^{1}H NMR spectrum (600 MHz) of compound **1** in $CDCl_3$.

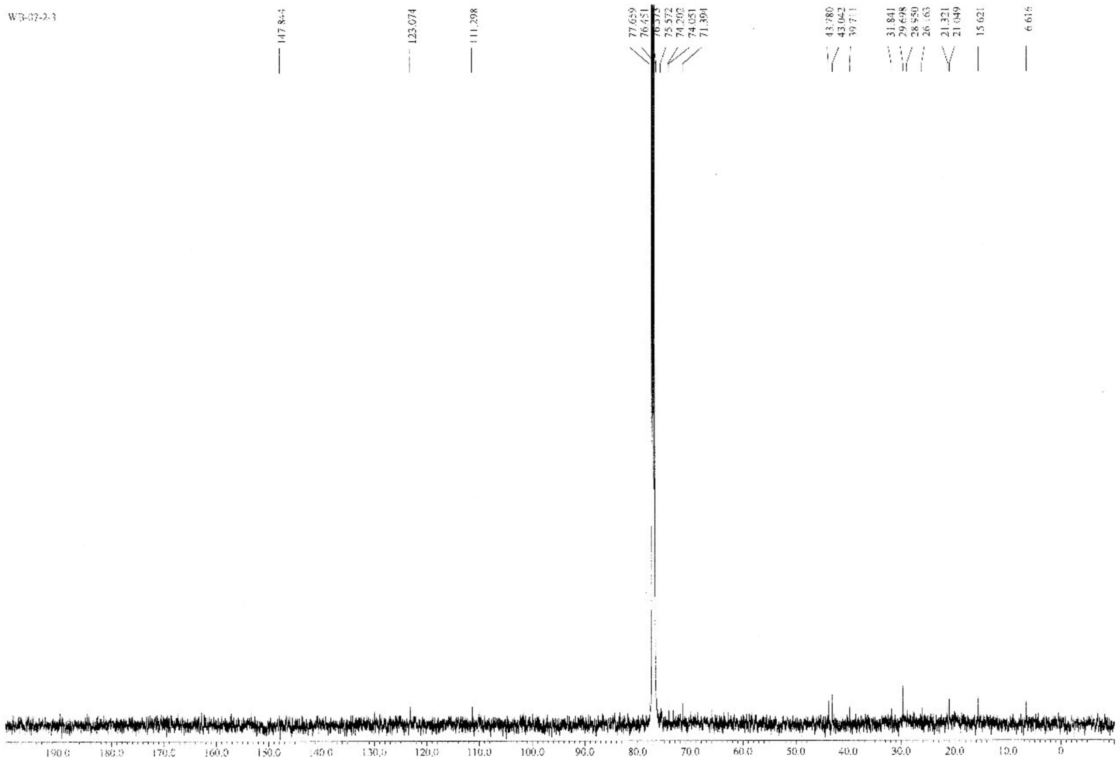

S5. ^{13}C NMR spectrum (150 MHz) of compound **1** in CDCl3.

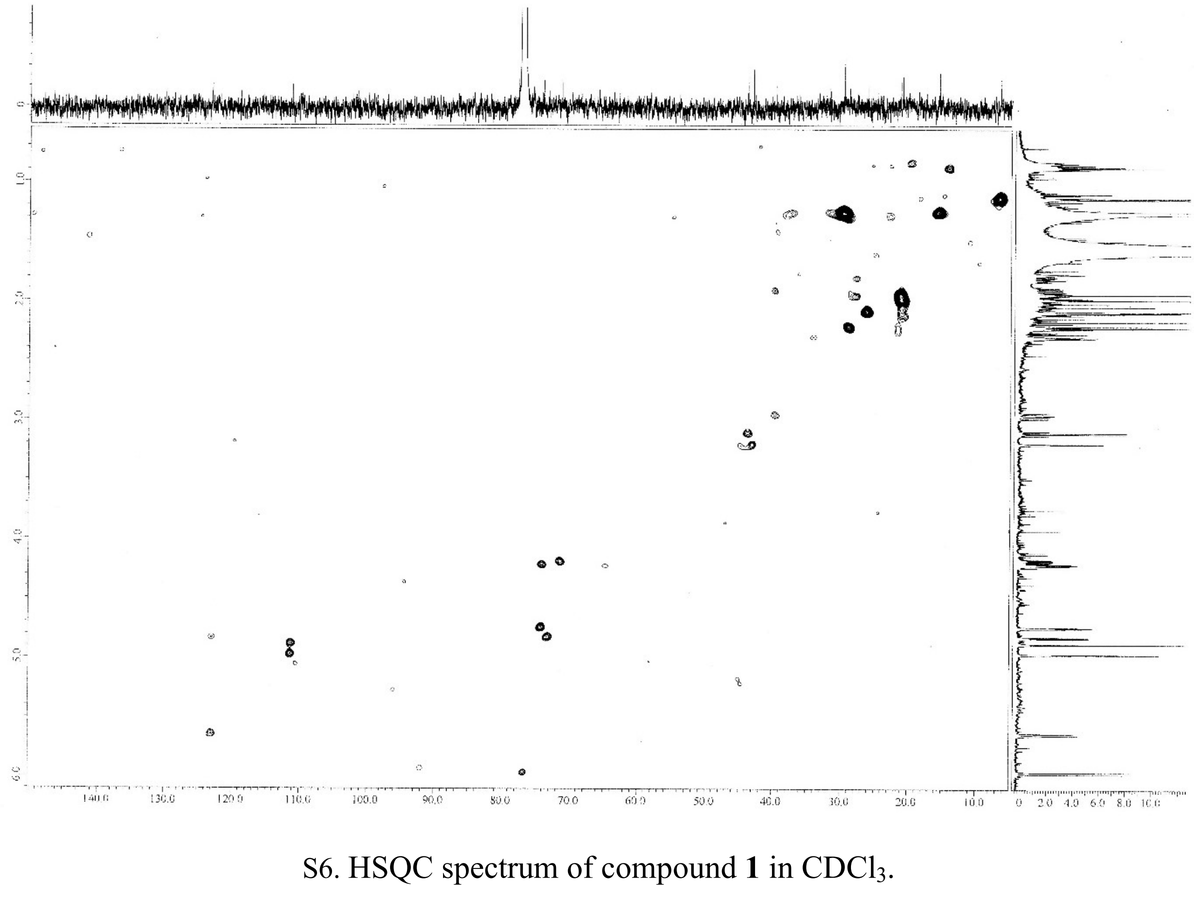

S6. HSQC spectrum of compound **1** in $CDCl_3$.

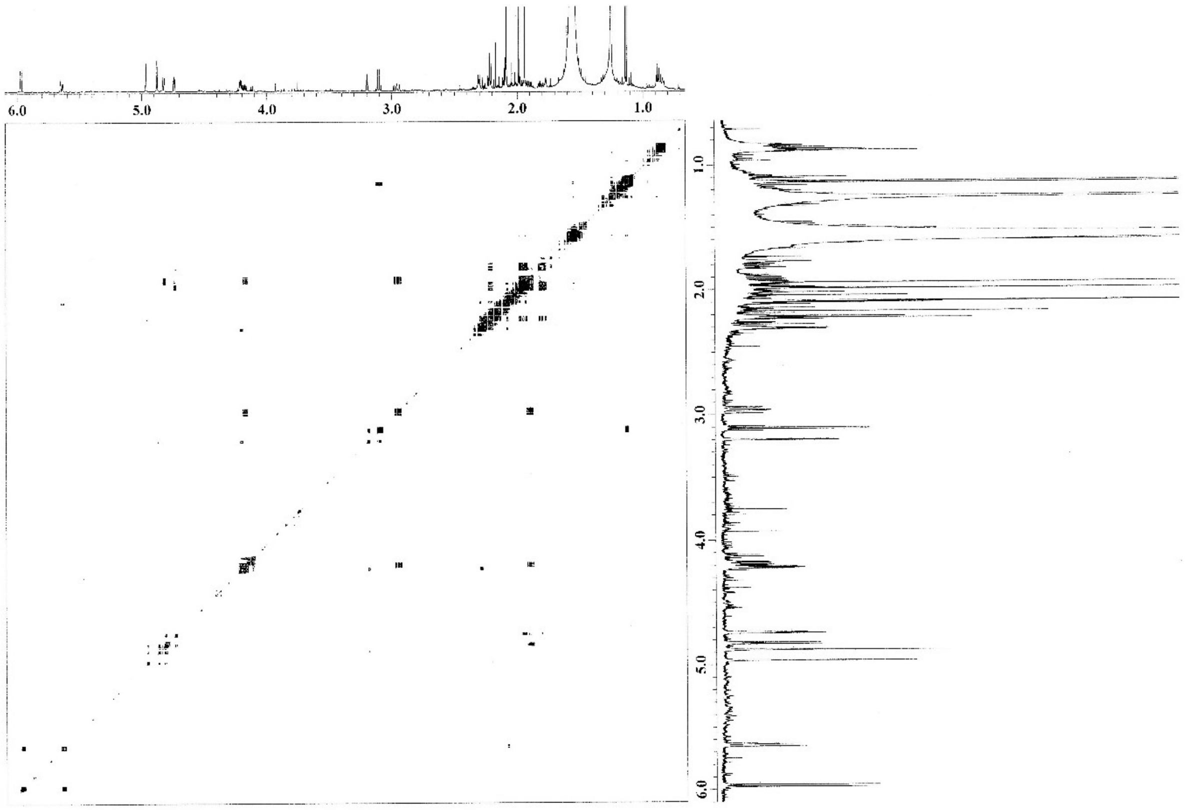

S7. $^{1}H-^{1}H$ COSY spectrum of compound **1** in $CDCl_3$.

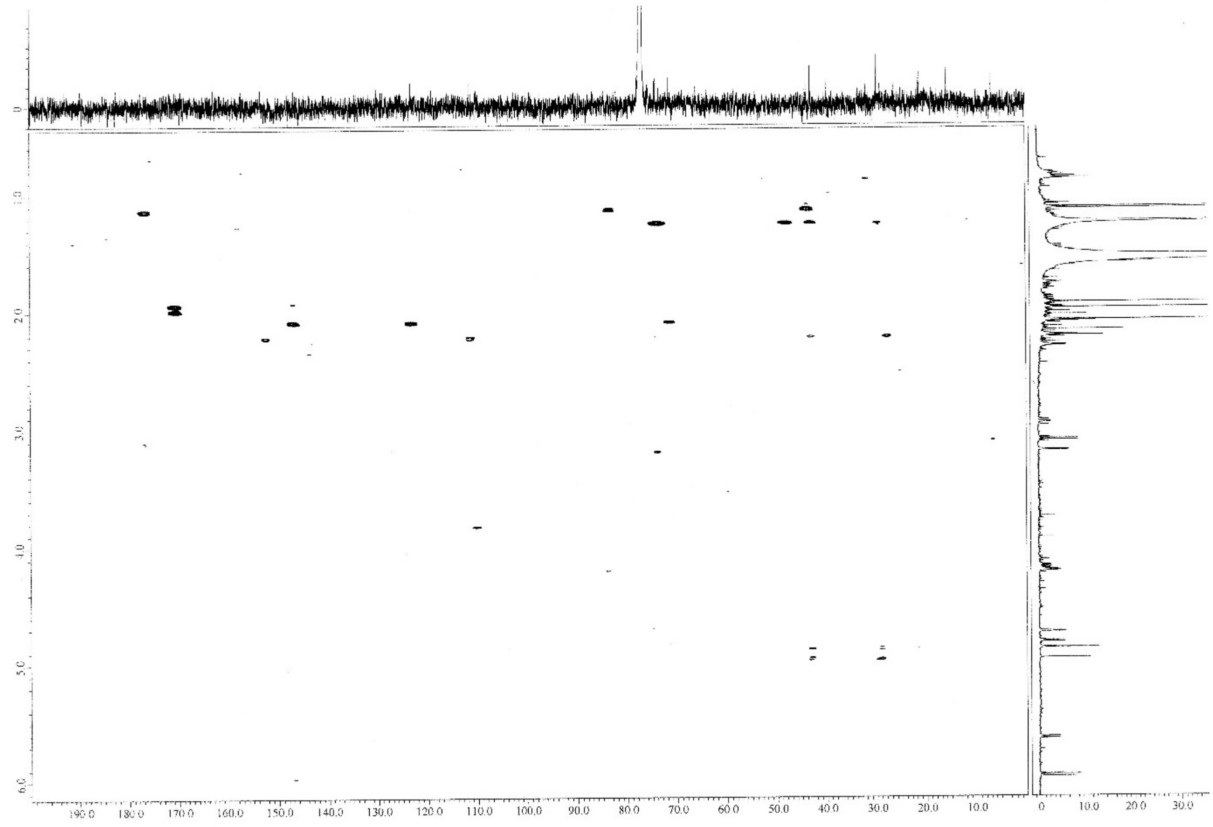

S8. HMBC spectrum of compound **1** in $CDCl_3$.

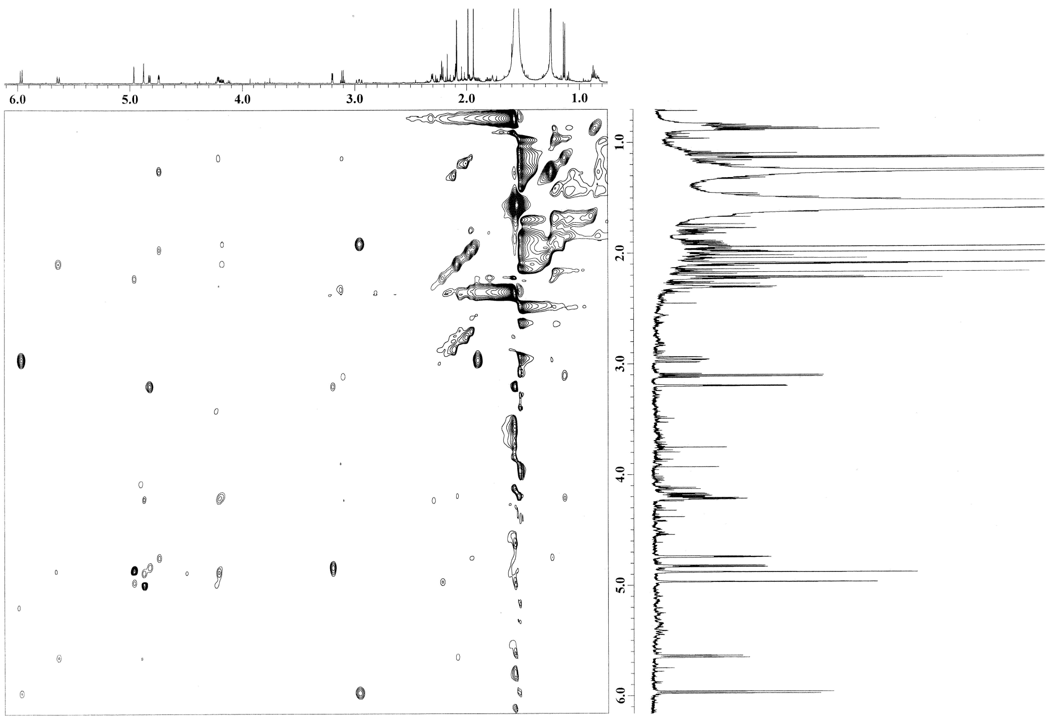

S9. NOESY spectrum of compound **1** in $CDCl_3$.

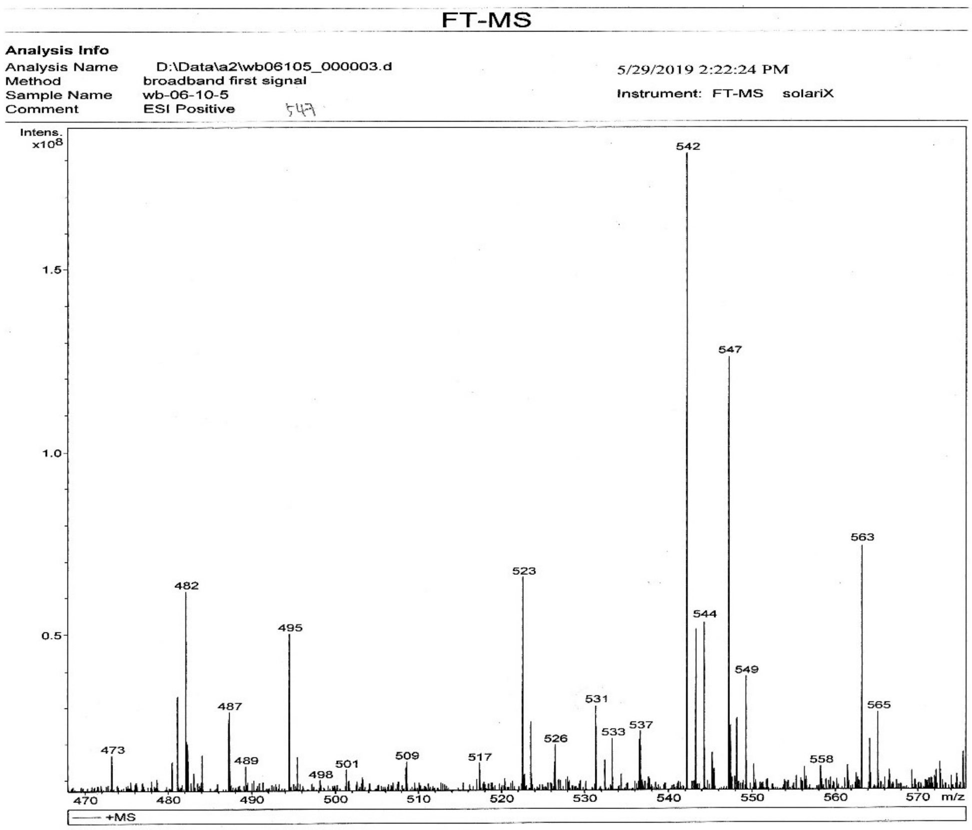

S10. ESIMS spectrum of compound **2**.

Mass Spectrum SmartFormula Report

Analysis Info

Analysis Name	D:\Data\a2\wb06105_000005.d	6/12/2019 4:13:22 PM
Method	broadband first signal	Operator: YU HSIAO-CHING
Sample Name	wb-06-10-5	Instrument: BRUKER FT-MS solariX
Comment	ESI Positive	

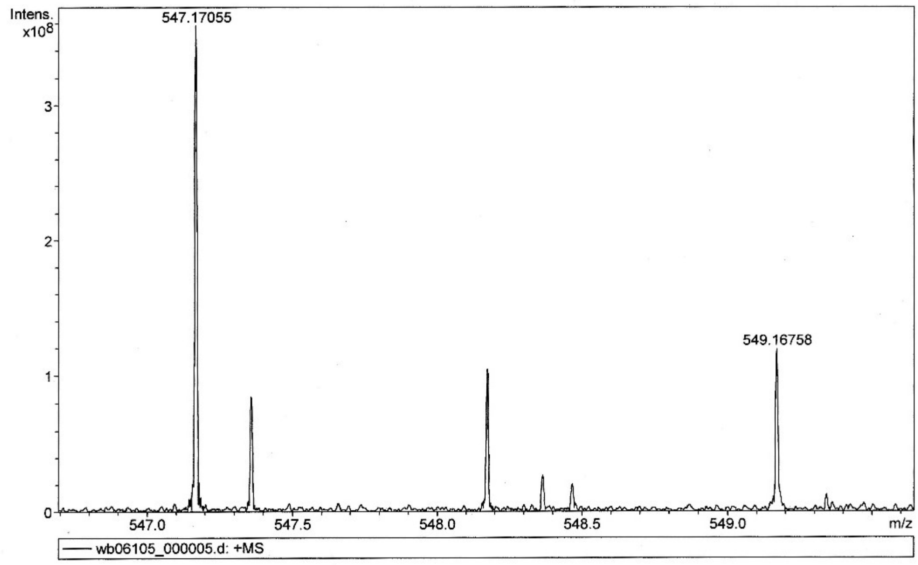

Meas. m/z	#	Formula	Score	m/z	err [mDa]	err [ppm]	mSigma	rdb	e⁻ Conf	N-Rule
547.17055	1	C 26 H 33 Cl Na O 9	100.00	547.17053	-0.02	-0.03	22.1	9.5	even	ok

S11. HRESIMS spectrum of compound **2**.

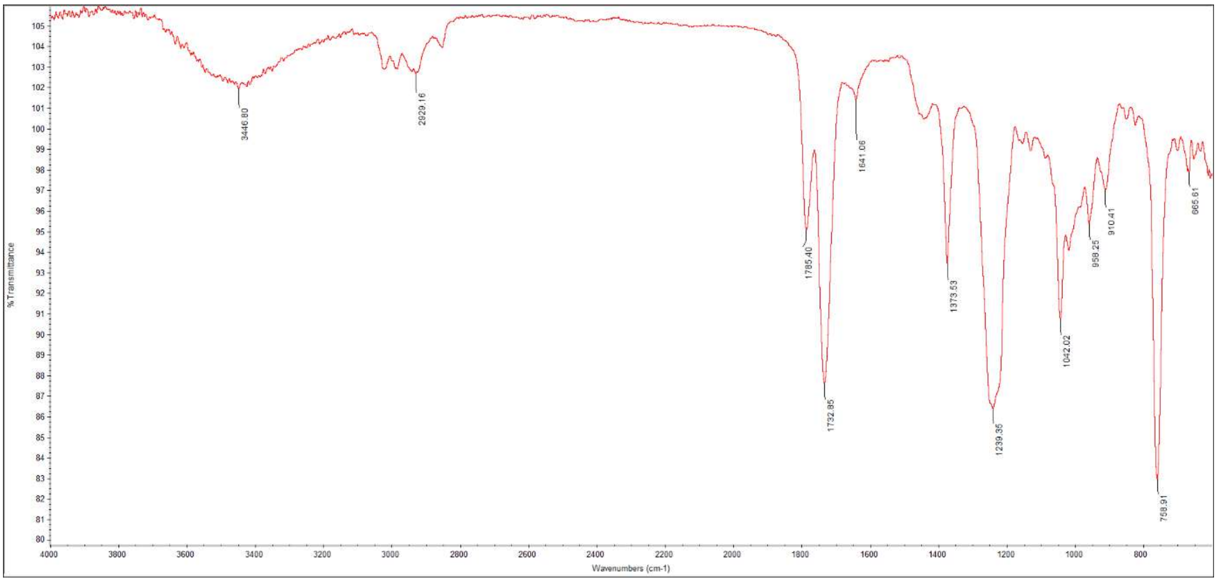

S12. IR spectrum of compound **2**.

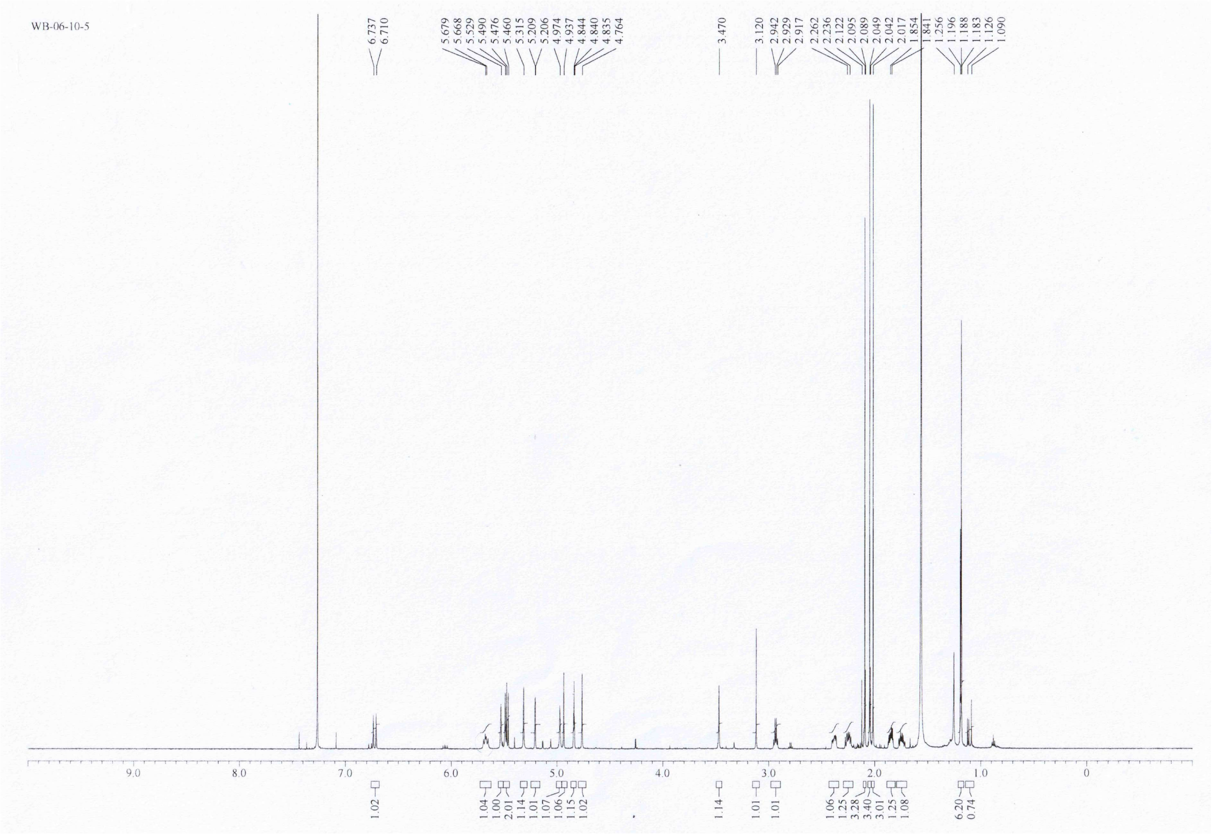

S13. ^{1}H NMR spectrum (600 MHz) of compound **2** in $CDCl_3$.

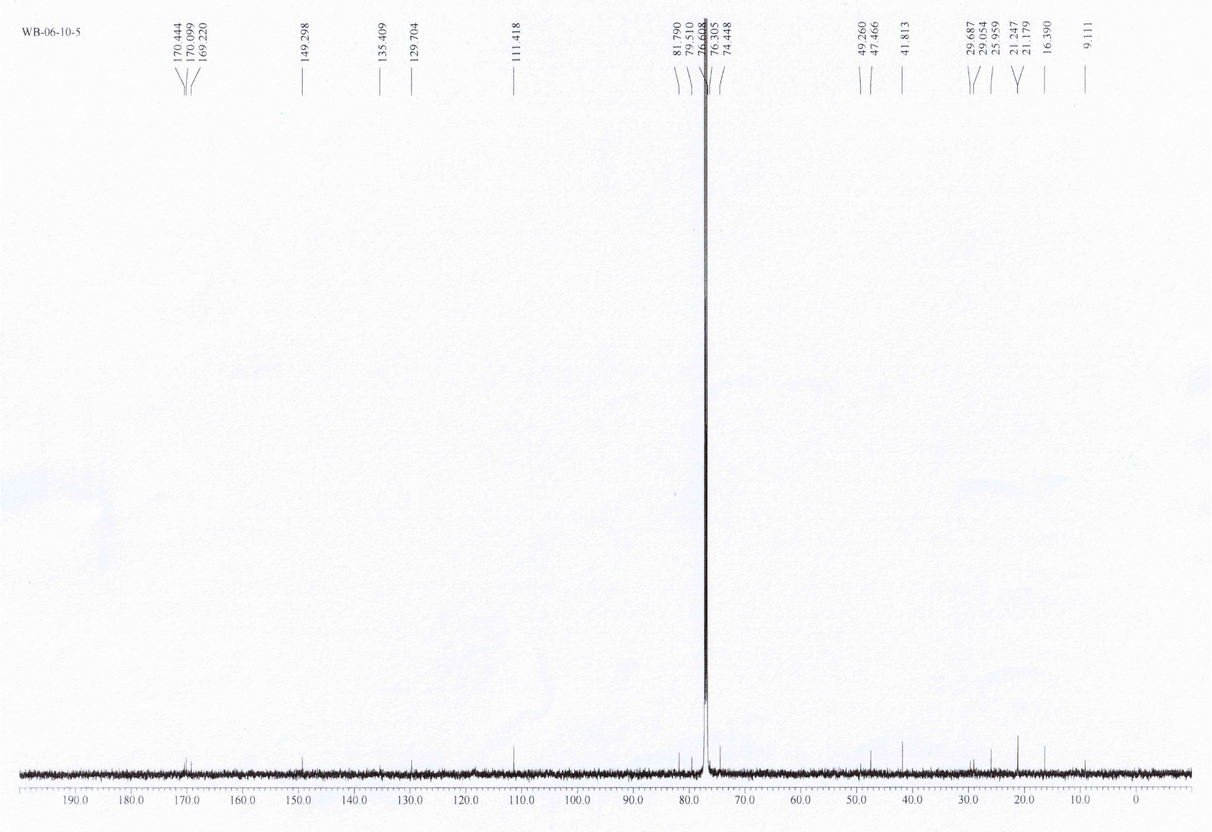

S14. ^{13}C NMR spectrum (150 MHz) of compound **2** in $CDCl_3$.

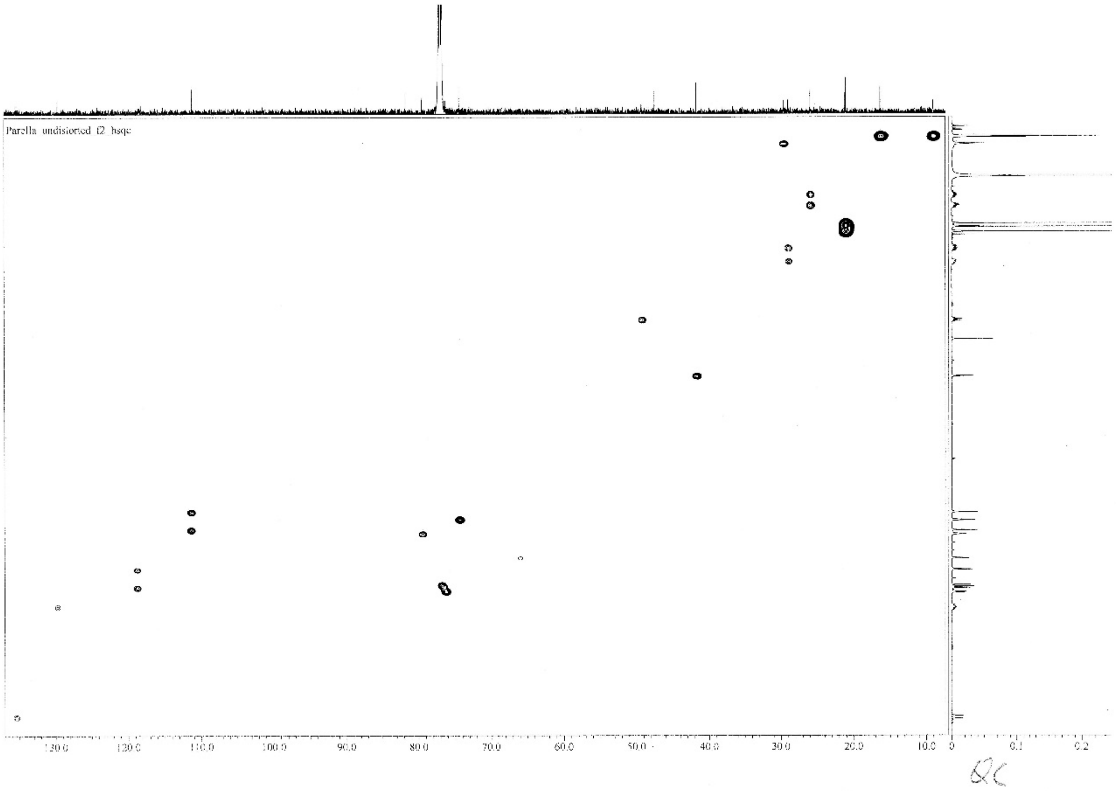

S15. HSQC spectrum of compound **2** in $CDCl_3$.

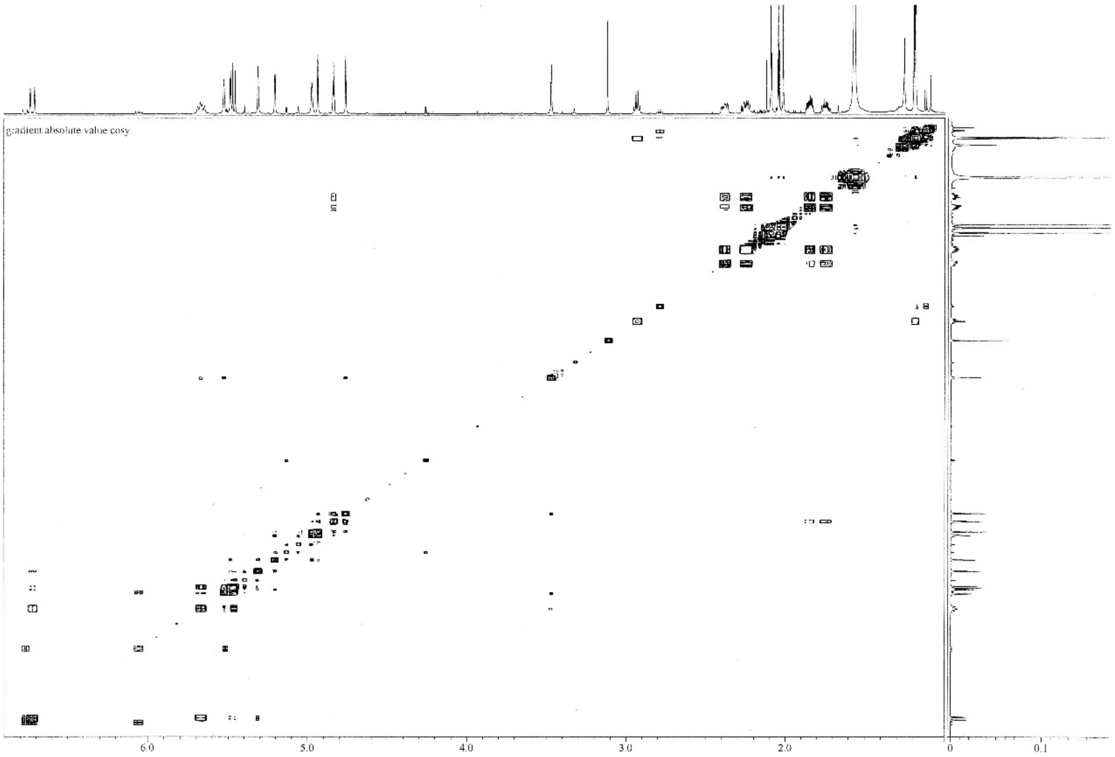

S16. ^{1}H–^{1}H COSY spectrum of compound **2** in $CDCl_3$.

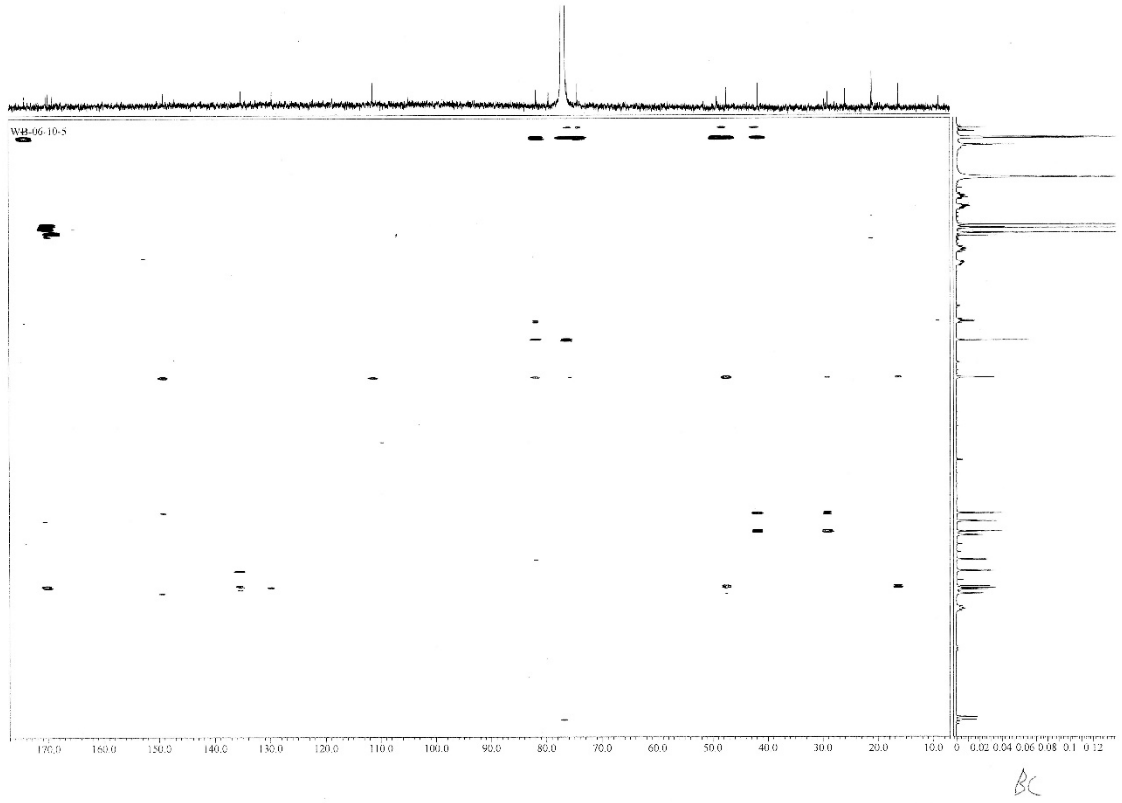

S17. HMBC spectrum of compound **2** in $CDCl_3$.

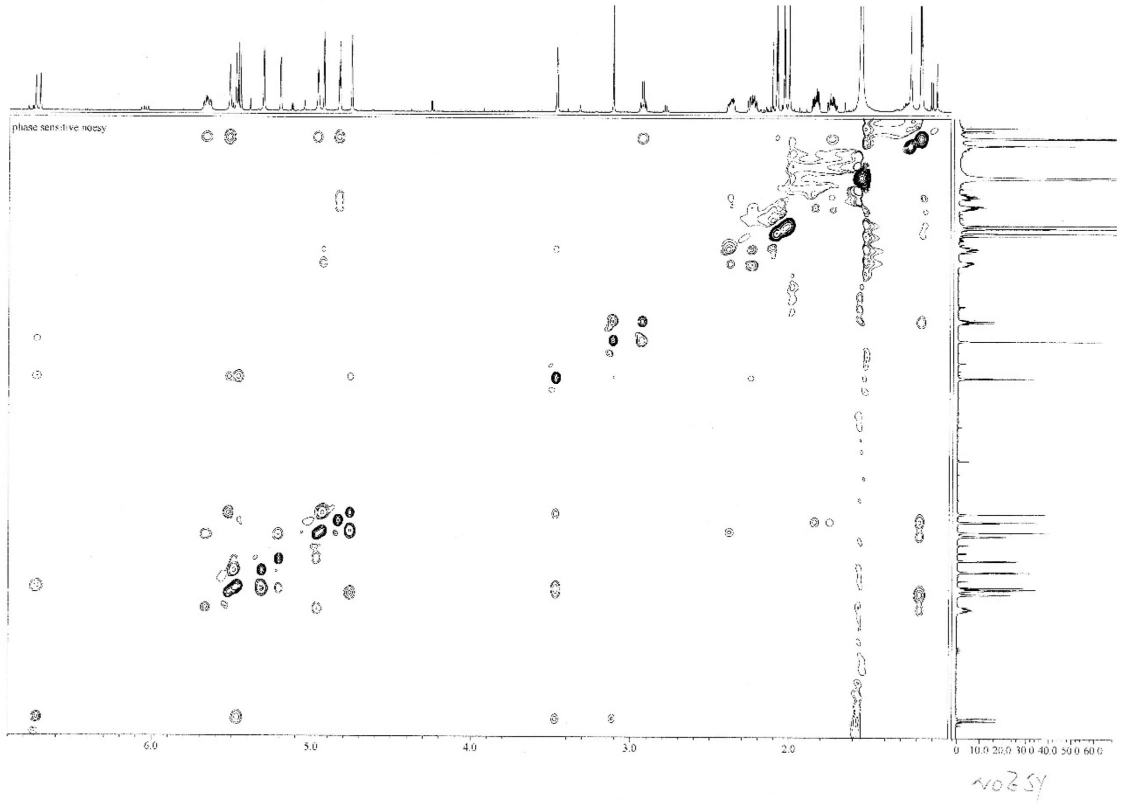

S18. NOESY spectrum of compound **2** in $CDCl_3$.

FT-MS

Analysis Info
Analysis Name D:\Data\a2\wb061011_000003.d
Method broadband first signal
Sample Name wb-06-10-11
Comment ESI Positive

5/29/2019 2:53:40 PM
Instrument: FT-MS solariX

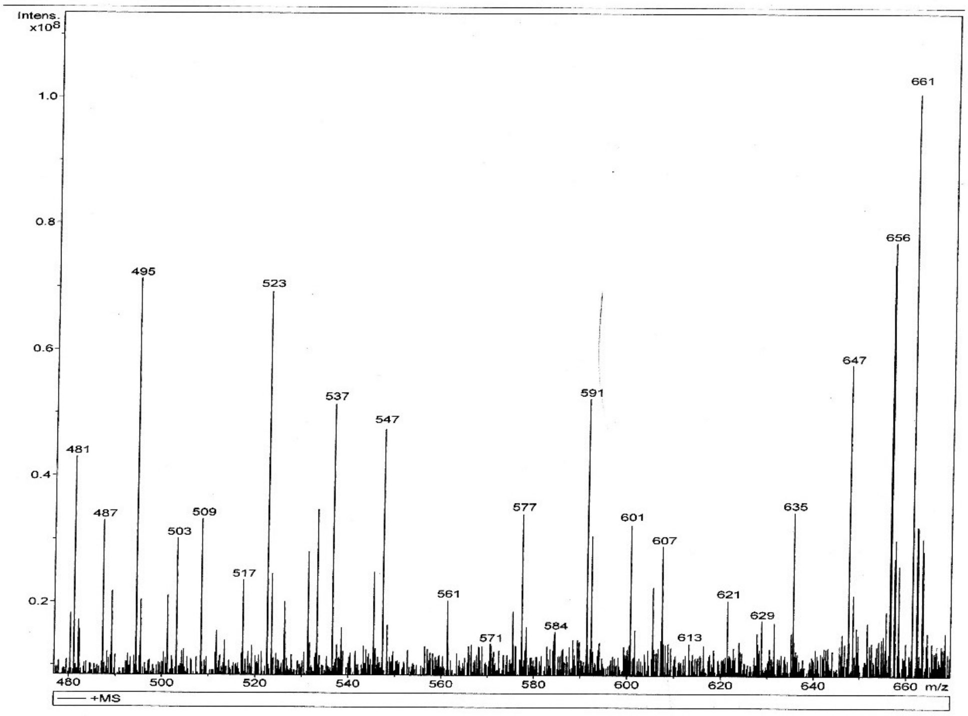

S19. ESIMS spectrum of compound **3**.

Mass Spectrum SmartFormula Report

Analysis Info
Analysis Name D:\Data\a2\wb061011_000005.d
Method broadband first signal
Sample Name wb-06-10-11
Comment ESI Positive

5/29/2019 2:52:59 PM
Operator: YU HSIAO-CHING
Instrument: BRUKER FT-MS solariX

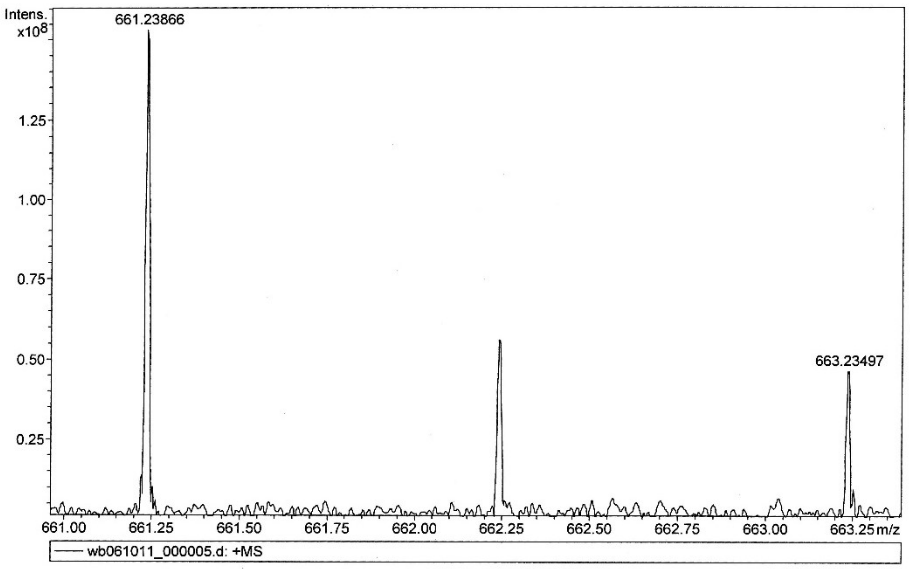

Meas. m/z	#	Formula	Score	m/z	err [mDa]	err [ppm]	mSigma	rdb	e⁻ Conf	N-Rule
661.23866	1	C 32 H 43 Cl Na O 11	100.00	661.23861	-0.05	-0.07	45.2	10.5	even	ok

S20. HRESIMS spectrum of compound **3**.

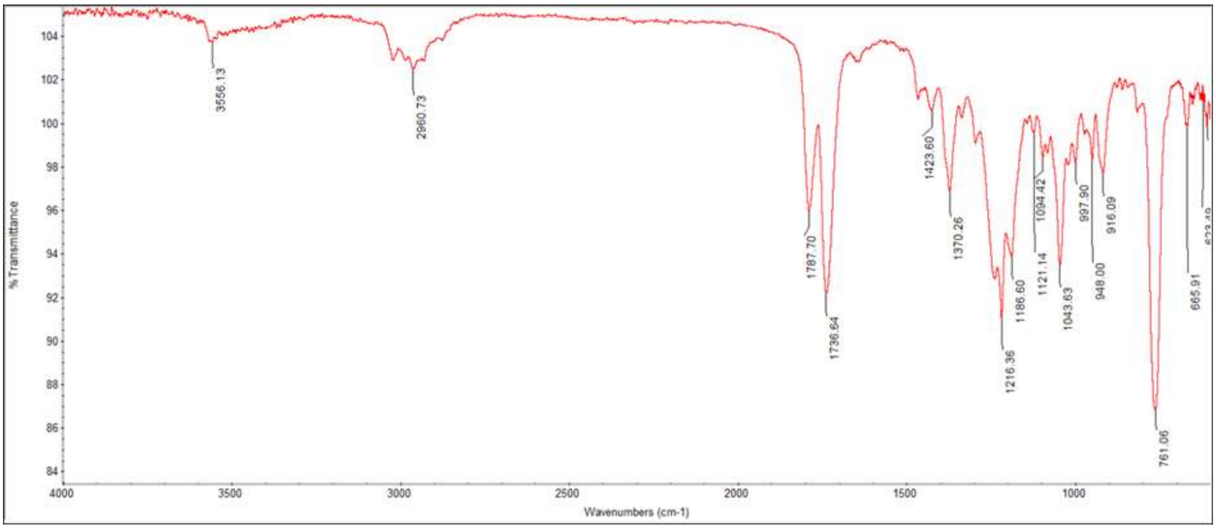

S21. IR spectrum of compound **3**.

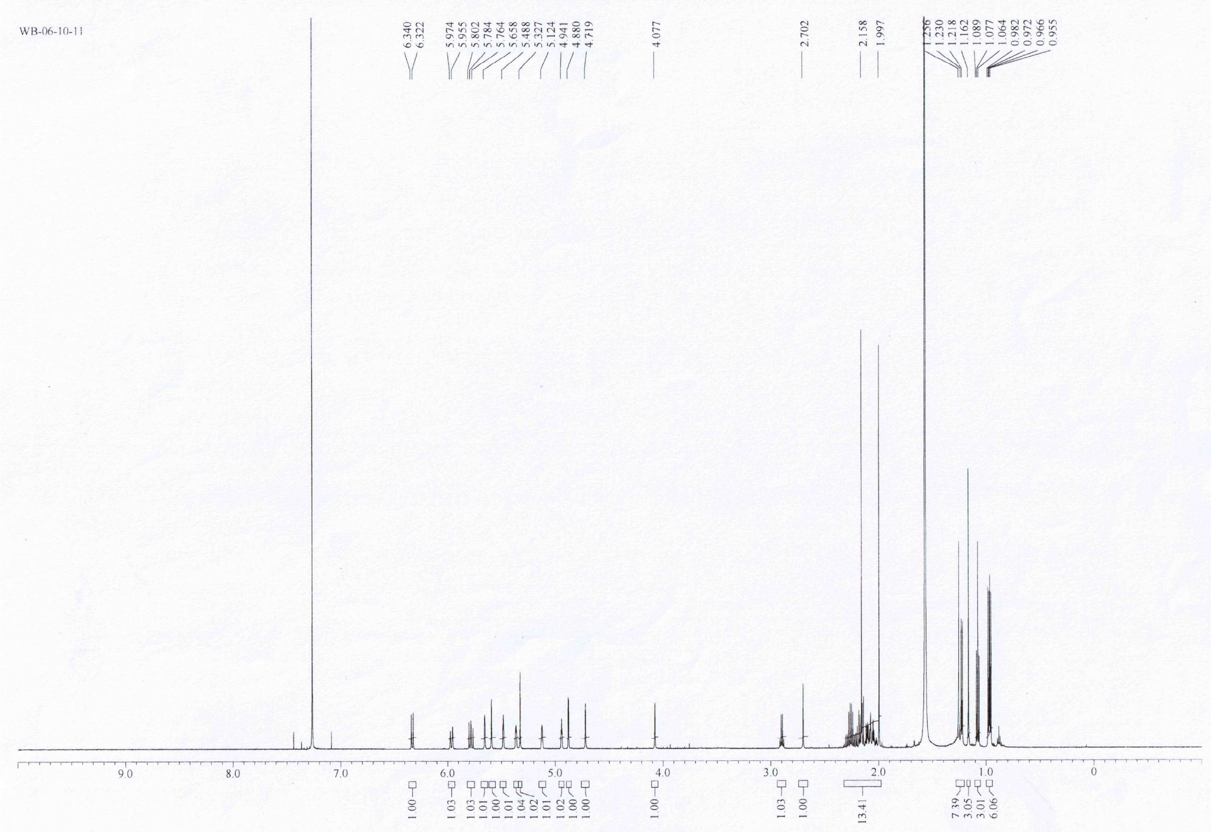

S22. ^{1}H NMR spectrum (600 MHz) of compound **3** in $CDCl_3$.

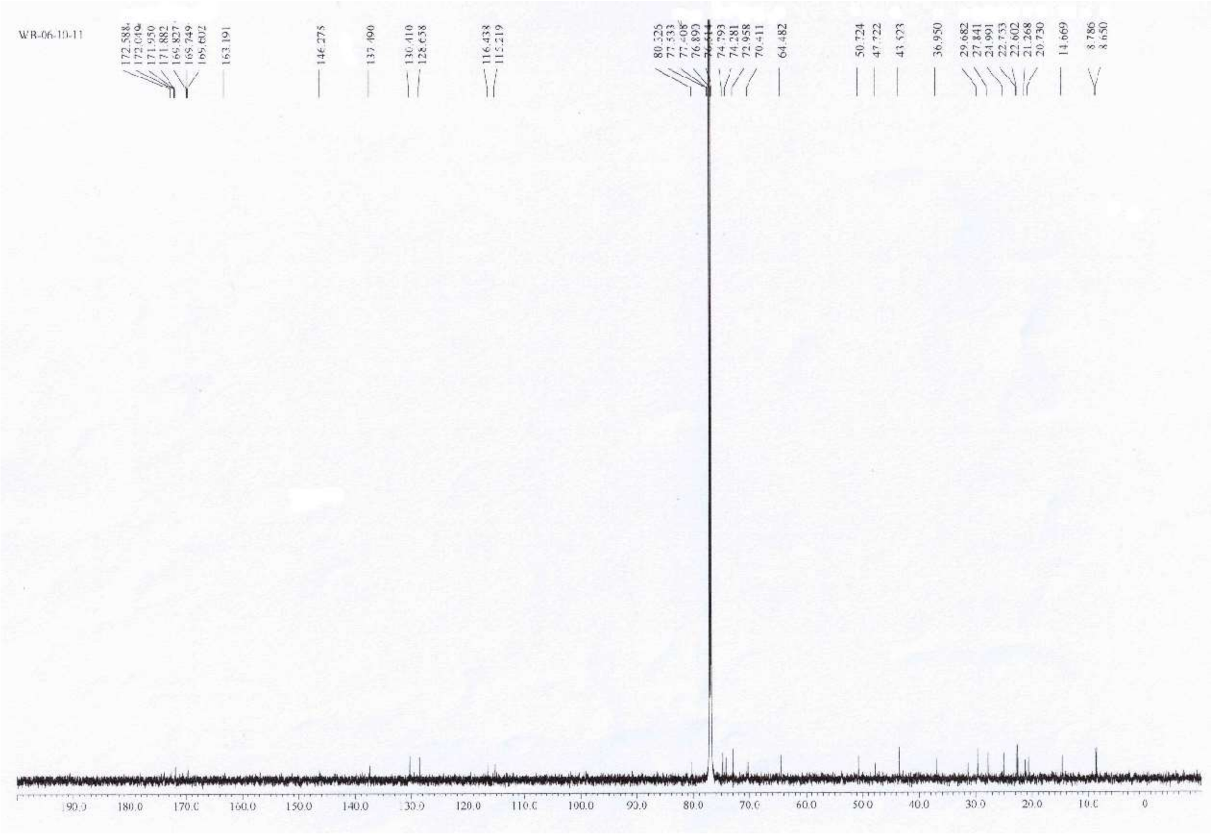

S23. ^{13}C NMR spectrum (150 MHz) of compound **3** in $CDCl_3$.

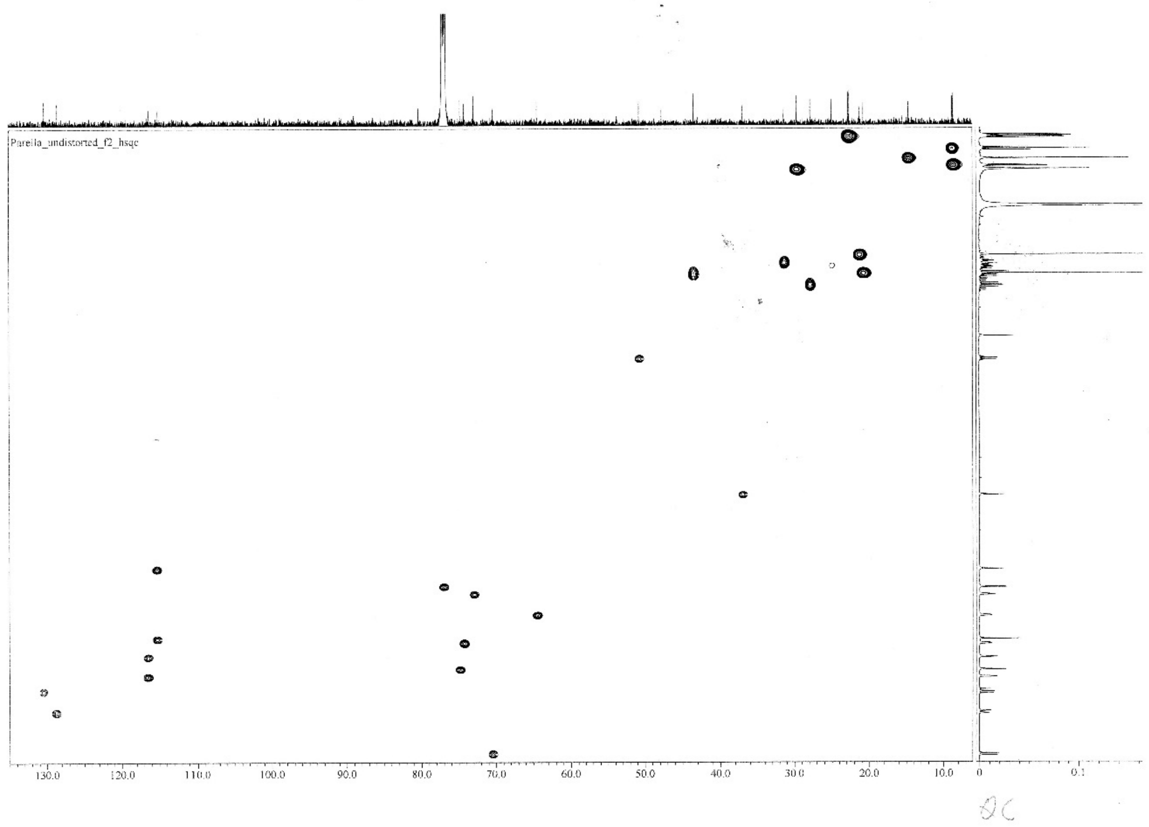

S24. HSQC spectrum of compound **3** in $CDCl_3$.

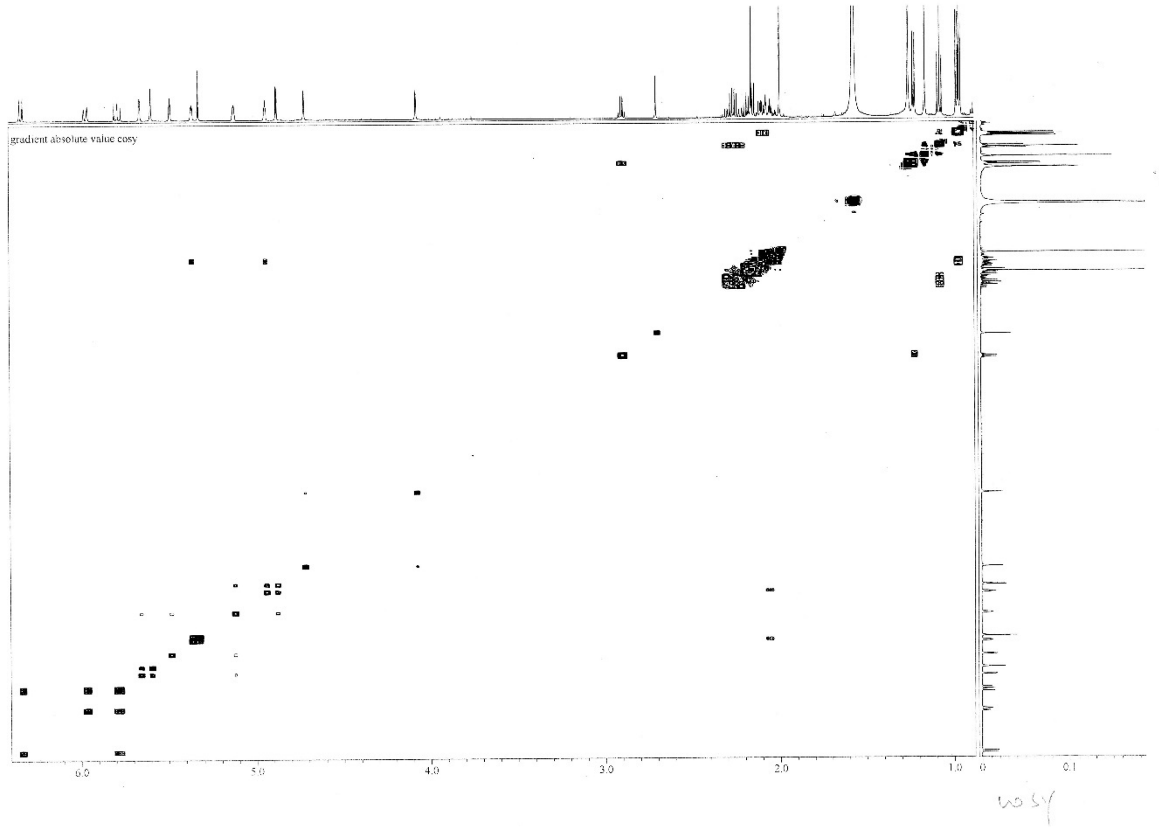

S25. ^1H–^1H COSY spectrum of compound **3** in $CDCl_3$.

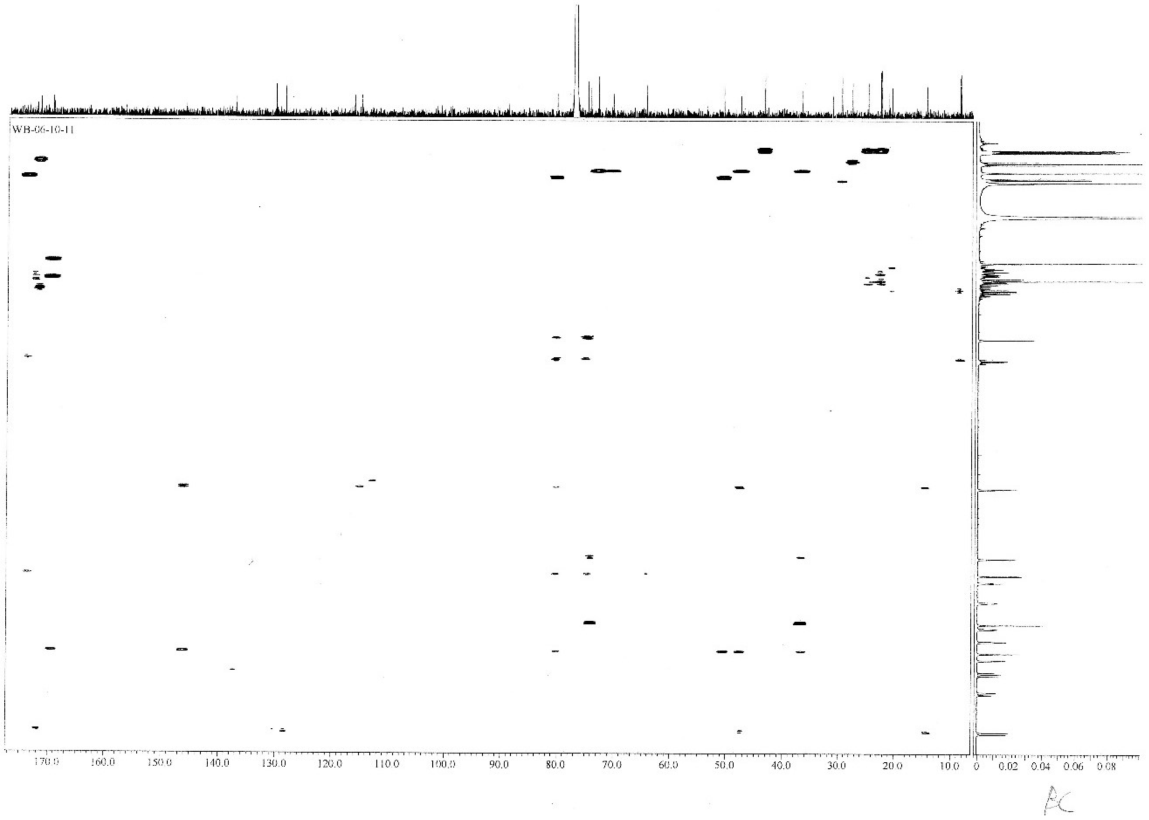

S26. HMBC spectrum of compound **3** in $CDCl_3$.

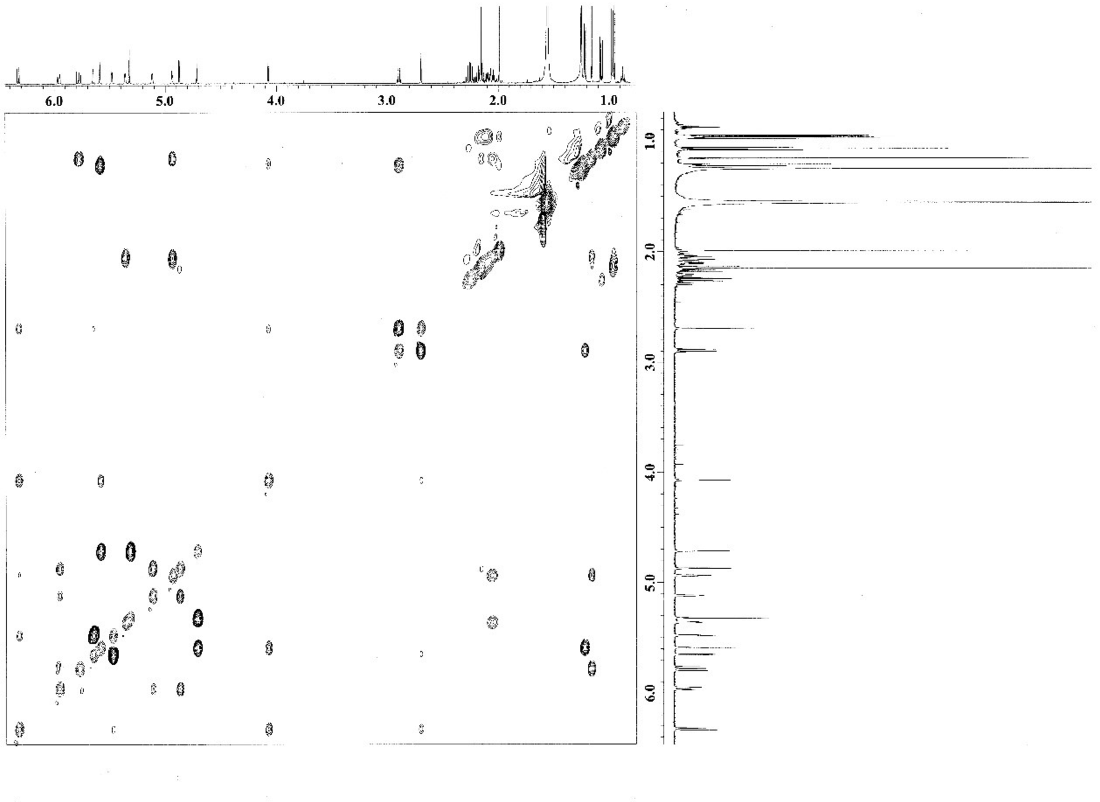

S27. NOESY spectrum of compound **3** in $CDCl_3$.
